# Partner similarity and social cognitive traits predict social interaction success among strangers

**DOI:** 10.1093/scan/nsad045

**Published:** 2023-09-12

**Authors:** Sarah L Dziura, Aditi Hosangadi, Deena Shariq, Junaid S Merchant, Elizabeth Redcay

**Affiliations:** Department of Psychology, University of Maryland, College Park, MD 20742, USA; Center for Mind and Brain University of California Davis, Davis, CA 95618, USA; Department of Psychology, University of Maryland, College Park, MD 20742, USA; Neuroscience and Cognitive Science Program, University of Maryland, College Park, MD 20742, USA; Neuroscience and Cognitive Science Program, University of Maryland, College Park, MD 20742, USA; Department of Psychology, University of Maryland, College Park, MD 20742, USA; Neuroscience and Cognitive Science Program, University of Maryland, College Park, MD 20742, USA

**Keywords:** social interaction, neural similarity, perceived similarity, social skills, empathy

## Abstract

Social interactions are a ubiquitous part of engaging in the world around us, and determining what makes an interaction successful is necessary for social well-being. This study examined the separate contributions of individual social cognitive ability and partner similarity to social interaction success among strangers, measured by a cooperative communication task and self-reported interaction quality. Sixty participants engaged in a 1-h virtual social interaction with an unfamiliar partner (a laboratory confederate) including a 30-min cooperative ‘mind-reading’ game and then completed several individual tasks and surveys. They then underwent a separate functional MRI session in which they passively viewed video clips that varied in content. The neural responses to these videos were correlated with those of their confederate interaction partners to yield a measure of pairwise neural similarity. We found that trait empathy (assessed by the interpersonal reactivity index) and neural similarity to partner both predicted communication success in the mind-reading game. In contrast, perceived similarity to partner and (to a much lesser extent) trait mind-reading motivation predicted self-reported interaction quality. These results highlight the importance of sharing perspectives in successful communication as well as differences between neurobiological similarity and perceived similarity in supporting different types of interaction success.

Interactions with others are an integral part of everyday life, occurring frequently and serving as the building blocks for relationship development among social groups (Baumeister and Leary, 1995; Seyfarth and Cheney, 2012). These interactions are critically important as forming and maintaining high-quality social relationships provide many long-term benefits, including greater happiness and support, as well as reduced risk for dementia and mortality (Holt-Lunstad *et al.*, 2010; Thoits, 2011; Kuiper *et al.*, 2015; Holt-Lunstad, 2018). Even among weaker social ties, regular interactions are beneficial and track positively with feelings of happiness and belonging (Granovetter, 1973; Epley and Schroeder, 2014; Sandstrom and Dunn, 2014). Thus, a thorough understanding of the factors that contribute to successful interactions among novel partners is needed. Both individual-level (e.g. social cognition) and dyad-level (e.g. partner similarity) factors may impact how well we communicate with others in a novel setting, as well as how successful we perceive such social interactions to be.

Social interactions are assumed to be strongly influenced by individual traits, such as social motivation, perspective-taking or empathy (Geen, 1991; Depue and Morrone-Strupinsky, 2005; Frith and Frith, 2006; Blanke and Riediger, 2019). Empirical findings reveal modest links between social cognitive traits and outcomes such as relationship quality and maintenance (Gleason *et al.*, 2009; Lecce *et al.*, 2017; Nilsen and Bacso, 2017; Sened *et al.*, 2017). However, there is a disconnect between examining social cognitive ability through trait assessments or laboratory tasks and how social interactions unfold naturally (Zaki *et al.*, 2008; Alkire *et al.*, 2023). Dynamic interactions provide rich verbal and non-verbal information directly to the interaction partner and in turn require reciprocal information from them. It is therefore necessary to consider not only the social ability of the individual but also dyadic factors resulting from pairing two individuals together (Redcay and Schilbach, 2019; Wheatley *et al.*, 2019; Kingsbury and Hong, 2020).

Research has demonstrated the importance of dyadic factors, such as existing inter-individual similarity (i.e. homophily) and socially constructed similarity (i.e. conformity), in predicting social interaction success. Partners synchronize body movements while interacting (Bernieri and Rosenthal, 1991; Palumbo *et al.*, 2017), and greater synchrony is associated with increased cooperation during social interactions (Cui *et al.*, 2012; Pan *et al.*, 2017; Reindl *et al.*, 2018; Nguyen *et al.*, 2020). This behavioral and physiological coordination is also linked to neural synchrony, which suggests greater understanding among communicating partners (Dumas *et al.*, 2010; Holper *et al.*, 2012; Jiang *et al.*, 2012; Yun *et al.*, 2012; Schoot *et al.*, 2016; Djalovski *et al.*, 2021). Recent studies have revealed distal links between neural similarity and social relationships as well; in particular, close relational ties show greater neural synchrony while interacting when compared to strangers (Kinreich *et al.*, 2017; Pan *et al.*, 2017; Reindl *et al.*, 2018; Djalovski *et al.*, 2021), and friends show more similar neural responses than indirect social network ties even when not interacting (Parkinson *et al.*, 2018). This propensity toward similarity reflects a fundamental way in which humans organize into social groups, as people are more likely to begin a relationship with an assumed similar stranger (Burger *et al.*, 2004; Guéguen *et al.*, 2011; Martin *et al.*, 2013). However, the involvement of individual traits in this process has not been well characterized. This study examined the effects of both individual and dyadic measures on two outcomes of interaction success: performance on a cooperative communication game and self-reported interaction quality.

Communication during a social interaction is suggested to follow a hierarchical pathway beginning with speech processing and the development of shared representations, leading to mutual understanding and finally resulting in relationship establishment and maintenance (Toni and Stolk, 2019; Wheatley *et al.*, 2019; Jiang *et al.*, 2021). This communication to achieve mutual understanding can be verbal or non-verbal; for example, gaze coordination is linked to mutual understanding and common ground knowledge (Shockley *et al.*, 2009); strangers can create shared representations of ambiguous signals through communication (Stolk *et al.*, 2014), and shared references lead to easier understanding between social partners (Nadig *et al.*, 2015). Theory suggests that this communicative process leads dyads to create a generalized shared reality that is not tied to specific topics of reference (Rossignac-Milon *et al.*, 2021). Importantly, however, these processes are not universal, as there are individual differences in the ability to converge on shared representational spaces (Wadge *et al.*, 2019; Wheatley *et al.*, 2019) and contextual factors that affect when and how these shared representations, or common ground, are used in the service of communication (Brown-Schmidt and Heller, 2018). For example, whether partners begin from closer or more distant mental representational spaces may impact creation of common ground and ultimately how successful the interaction is. Individuals with high social cognitive abilities may have an advantage in moving into this shared space, and interacting pairs who see and interpret the world more similarly may need to ‘travel’ less distance to reach mutual understanding. Additionally, those who are more similar may be able to rely on more ‘egocentric’ strategies without the need to enlist mentalizing abilities to achieve mutual understanding (Savitsky *et al.*, 2011). Examining these individual and dyadic factors in the context of performance on a cooperative communicative game is one way to shed light on these questions.

Successful communicative understanding is not the only important marker of a high-quality social interaction. People can enjoy playing a communication game together even if they do not end up performing well, or else, they can have other positive shared experiences that do not require mutual understanding but still result in social bonding. Feelings of partner closeness have been induced in strangers during single interactions through sharing personal information (Sedikides *et al.*, 2002), and self-reports of interaction quality are broadly associated with greater well-being, happiness and physiological functioning (Mote *et al.*, 2019; Sun *et al.*, 2020; Mauersberger *et al.*, 2022). Perceived interaction quality after the conclusion of a social interaction may therefore serve as an equally important independent marker of social interaction success.

In sum, both individual and dyadic mechanisms likely contribute to how successful a social interaction is. In this study, we examined the relationship between measures of social cognitive ability, similarity to partner and social interaction success. We hypothesized that both social cognitive ability and partner similarity would be positively related to interaction success, measured objectively by performance on a cooperative communication game and subjectively by self-reports of perceived interaction quality. We also hypothesized that similarity would moderate the relationship between social cognitive ability and interaction success, such that there would be a stronger relationship between social cognitive ability and interaction success in less similar partners, whereas more similar partners would have successful and high-quality interactions irrespective of individual social cognitive ability.

## Methods

The hypotheses and analysis plan for the data collected in this study were pre-registered on the Open Science Framework: https://osf.io/f3nu7. Amendments to the planned methods and analyses are explained in each section.

### Participants

Eighty-six adults (gender = 18 male, 66 female, 2 non-binary (NB); age range = 18–30 years; mean age = 21 years; race/ethnicity in Table 1) participated in the behavioral session. A subset of 60 participants participated in the functional magnetic resonance imaging (fMRI) session (gender = 12 male, 46 female, 2 NB; age range = 18–30 years; mean age = 21.1 years; race/ethnicity in Table 1). A further 14 participants were consented but did not ultimately complete either session due to MRI ineligibility or inability to commit to the second session. All participants signed an informed consent form in accordance with the Declaration of Helsinki and the Institutional Review Board at the University of Maryland and were compensated for their time through money or course credit. All second session participants were right-handed fluent English speakers with normal or corrected-to-normal vision. A sample size of 60 was noted in the pre-registration and budgeted for in the grant, as this would allow us to detect medium-size correlations (i.e. *r* = 0.35) with a power of 0.8. A minimum of 40 participants has previously been recommended for assessing group-level contrast effects (Geuter *et al.*, 2018). Thus, while we did not conduct a power analysis specific to the analyses within this paper, we were guided by a priori sample specification within funding and time constraints. The results presented in this paper are limited to data from the 60 participants who completed both sessions.

**Table 1.** Reported race/ethnicity of participants

Race/ethnicity category	Completed first session	Included in sample
American Indian/Alaskan Native	0 (0%)	0 (0%)
Asian	22 (25.58%)	12 (20%)
Black or African American	17 (19.77%)	8 (13.33%)
Hispanic or Latino	7 (8.14%)	5 (8.33%)
Native Hawaiian or Other Pacific Islander	1 (1.16%)	1 (1.67%)
White	31 (36.05%)	26 (43.33%)
More than one race	5 (5.81%)	5 (8.33%)
Others, unknown, unreported	3 (3.49%)	3 (5%)
Total	86	60

### Behavioral session

Participants completed a 1-h virtual interaction over Zoom (Zoom Video Communications Inc, 2016) with a partner who they believed to be another study participant. This partner was an undergraduate laboratory confederate trained to engage in these interactions naturally, while giving consistent responses across sessions. A total of eight confederates were used across the entire experiment, and the effect of confederate (i.e. which partner the participant was paired with) was included in all analyses. The pair completed three semi-structured introduction and discussion tasks where confederates gave similar answers to the participant, but conversation was otherwise allowed to flow naturally. These consisted of (i) 5 min of unstructured conversation to introduce themselves, (ii) creating a joint ‘top five list’ from a selection of categories (movies, TV shows, books and musicians) and (iii) asking and answering seven discussion questions. We describe these activities in Supplementary Methods in Supplementary Data for context, but no analysis was conducted specifically on these tasks. Participants then played a ‘mind-reading’ game to assess cooperative communication success. Following the interaction, participants completed a 1-h individual virtual session comprising performance tasks and surveys.

#### Social interaction success measures

Communicative success was measured through playing Telewave, an online cooperative ‘mind-reading’ game (https://github.com/gjeuken/telewave). One person received a prompt on a binary scale (e.g. Hot–Cold) and was secretly shown a bullseye correct answer between 0 and 100 (which their partner did not see). They then gave a clue to their partner, who tried to guess where the bullseye fell between the two extremes. For the ‘Hot–Cold’ example, if the bullseye was slightly to the left of center, a clue might be ‘salad’ because it is a little colder than room temperature. After 15 rounds, the roles were reversed, but only the trials in which the participant was the guesser were analyzed. They were told that the goal was to work together to get as close to the bullseye as possible. Success was measured as the numerical distance between the participant’s guess and the correct bullseye, averaged over all trials. All trials were identical across participants, and confederates gave the same clues; these clues had been developed organically by confederates during study design. Some trials were specifically designed to reference topics of conversation that partners may have engaged in during the introduction tasks. Extended details can be found in Supplementary Methods in Supplementary Data.

Perceived interaction quality was operationalized as the sum of three questions (‘How well did your interaction with your partner go overall?’, ‘How much did you enjoy your interaction with your partner overall?’ and ‘How much would you want to continue a friendship with your partner after the experiment?’). Questions were given on a five-point Likert scale from ‘Not at all’ to ‘The Most’. A composite sum of overall quality rather than separate items was used, as this allowed for a greater range of responses than the original scales. These questions were answered via Qualtrics immediately following the interaction task, after the participant and a session administrator went into a separate virtual room.

#### Social cognitive measures

- Complex Emotion Recognition in Faces Task (Golan *et al.*, 2006), which assesses the ability to identify complex emotions on brief silent videos of a variety of faces. Performance was calculated as percent accuracy out of 50 trials.
- Empathic Accuracy from Emotional Narratives Task (Ong *et al.*, 2021), which assesses the ability to track positive and negative emotional valence during an audiovisual narrative. Performance was calculated by correlating subject ratings and ratings gathered from the story narrator after the video was recorded.
- Interpersonal Reactivity Index (Davis, 1980, 1983). This measure assesses the reactions of an individual to the observed experiences of others. The sum total of all items was used as a measure of global empathy.
- Mind-Reading Motivation Scale (Carpenter *et al.*, 2016). This measure assesses an individual’s propensity toward engaging with others’ perspectives and mental states.

We use ‘social cognitive’ throughout the manuscript as a way to distinguish these individual measures from the dyadic similarity measures, although they include social-emotional and empathic as well as mentalizing traits.

#### Similarity measures

- Big Five Personality Trait Mini-Markers (Saucier, 1994). Item-level Pearson correlations were conducted between participant responses and their interaction partner to yield a measure of personality similarity.
- Modified Avocation Activities Questionnaire (McManus *et al.*, 2011). This measure assesses the frequency of engaging in leisure activities outside of work or school. Some items were modified to include newer activities that students are more likely to engage in (Supplementary Materials in Supplementary Data). Item-level Pearson correlations were conducted between the participant and the interaction partner to yield a measure of similarity in interests and activities.
- Perceived Similarity. Participants were asked on a five-point Likert scale to assess how similar they felt to their partner (‘Overall, how similar are you and your partner?’).

### fMRI session: neural similarity

#### Task design

Participants passively viewed a series of naturalistic videos that simulated the feeling of ‘channel surfing’ on television (Supplementary Table S2). Twelve video clips, each 2- to 5-min long, were presented with audio to the participants across four 10-min runs. These clips covered a variety of scenarios (e.g. comedy shows, documentaries, cooking shows and reality television). A similar task using a different set of videos has been shown to elicit neural similarity that tracks with friendship (Parkinson *et al.*, 2018).

#### fMRI image acquisition and processing

fMRI data were collected on a 3.0 Tesla Siemens MAGNETOM scanner with a 32-channel head coil. Halfway through data collection, the scanner was upgraded from a Trio Tim to a Prisma Fit system (*n*_Trio_ = 27, *n*_Prisma_ = 33). Scan parameters were kept largely identical, and neural similarity was only calculated among partners scanned on the same system (e.g. confederates scanned on a Trio would not be paired with a participant with a Prisma scan). Visual stimuli were presented on a rear projection screen and viewed by participants on a head coil–mounted mirror. Audio was presented to participants through memory foam earbuds (Canal Tips, complyfoam.com), which provide sound isolation and noise-canceling properties for hearing protection; audio volume was tested and adjusted prior to the scan onset for each participant’s hearing and comfort. One subject’s scan was stopped due to discomfort, and four subjects were excluded for falling asleep during more than two runs of the task. Twelve additional subjects had partially poor (e.g. falling asleep) data in two or fewer runs, and those individual runs were removed from analysis before correlation with their partner. Six subjects had one run removed (resulting in 27.4 included minutes of data), and six subjects had two runs removed (resulting in 18.3 included minutes of data).

Preprocessing was conducted in fMRIprep 20.2.5 (Esteban *et al.*, 2018). Briefly, functional data were skull-stripped, fieldmap-corrected to minimize susceptibility distortion, slice-time corrected, registered first to the T1-weighted anatomical scan and then resampled to standard Montreal Neurological Space. Motion artifacts were identified and removed through independent component analysis (ICA), specifically ICA-AROMA (ICA-based Automatic Removal of Motion Artifacts), non-aggressive option), and data were spatially smoothed by a 6-mm full width half maximum Gaussian kernel. As motion was minimal across participants, additional motion regression beyond ICA removal was not conducted. Similarity in framewise displacement (FD) across volumes among partners was calculated and added as a covariate in all group-level analyses that included neural similarity. Preprocessing details directly from the fMRIprep output and supplementary motion effects analyses can be found in Supplementary Methods in Supplementary Data.

After preprocessing with fMRIprep, the BOLD signal from each run was masked to remove non-brain signals and scaled to percent signal change. Each run was trimmed by 21 initial volumes, and volumes beyond the end of each final video were discarded, consistent with recommendations for inter-subject correlation analysis with naturalistic videos (Nastase *et al.*, 2019). Runs were concatenated for extraction from a 268-region atlas defined from resting-state functional data (Shen *et al.*, 2013; Finn *et al.*, 2015). This atlas was chosen over the Desikan–Killiany anatomical parcellation output from FreeSurfer for two reasons: first, functionally defined regions are likely to yield more accurate grouping of voxel values from naturalistic tasks over longer timescales; second, a greater number of regions allow grouping of data across fewer voxels, reducing the likelihood of smoothing out smaller region effects.

The final time course was averaged across non-zero voxels within each region. Pairwise Pearson correlations were calculated with these timecourse data between each participant and their confederate partner, and correlation values were averaged across all brain regions to yield a single measure (whole-brain similarity) for each participant. Neural similarity weighted by parcel size was also calculated but did not change the results (Supplementary Results in Supplementary Data). As we employed a new set of confederates after the scanner upgrade, no pairwise correlations were conducted across scanner types and all variance associated with the scanner upgrade was captured in the models by including the effect of the confederate partner.

### Data analysis

#### Model comparison

We used Bayes factor (BF) model comparisons to determine which measures were most likely to contribute to the success outcomes. BFs provide some advantages over frequentist statistics (Kruschke and Liddell, 2018) and quantify evidence for or against the model in the form of likelihood ratios without specific significance threshold cutoffs. For example, a BF of 10/1 indicates that the data are 10 times as likely under the proposed hypothesis compared to the alternative. The generalTestBF function with its default prior from the BF R package was used (Morey and Rouder, 2018) to conduct an all-possible-subset regression over 500 000 Monte Carlo iterations, resulting in a BF for each possible model compared to the null (intercept-only) model. The default Jeffreys–Zellner–Siow prior is centered around 0 and scaled at *r* = √2/4 and is generally accepted as a reasonable fit for data observed in psychological studies (Liang *et al.*, 2008; Rouder and Morey, 2012). To confirm that this choice did not affect our results, we conducted sensitivity analyses varying the prior distribution (Supplementary Tables S17–S20). Tests were conducted separately for social cognitive and similarity measures on each outcome (cooperative success and perceived interaction quality) and always included a between-subjects categorical variable to explain the additional effect of confederate. Models which included neural similarity as a predictor also included the number of runs of data available for each subject and motion similarity to partner as covariates of no interest. As this model comparison only works on complete datasets, subjects with missing measures were removed before each test was conducted. The measure of interest (i.e. not covariates) from each category with the highest BF was included in the follow-up interaction analysis.

#### Model interpretation

Generally accepted guidelines suggest the following interpretation of BF values: 1–3 = little or anecdotal evidence, 3–10 = moderate evidence, 10–100 = strong evidence and > 100 = extreme evidence (Lee and Wagenmakers, 2013). As the effect of the confederate partner was included in all models along with additional covariates associated with MRI data in all models including neural similarity, we further calculated relative BF values for how much more likely the model explains the outcome over the effect of these covariates of no interest (BF_model + cov/_BF_cov_).

## Results

### Communicative success

#### Social cognitive ability

The social cognitive measure comparison (*n* = 58) revealed that total scores on the interpersonal reactivity index (IRI) were the most predictive measure of communicative success (BF = 5.30) followed by total scores on the Mind-Reading Motivation Scale (MRMS) (BF = 3.84). Both measures reached the level of moderate evidence; however, the effect of confederate alone proved to be the best model (BF = 6.67) (Supplementary Figure S3; Supplementary Table S3). As the total IRI score was the strongest social cognitive measure, it was chosen for the follow-up interaction analysis. Exploratory analyses on IRI subscales revealed that this effect is driven by emotional empathy; these results can be seen in Supplementary Figures and Tables S10 and S11.

#### Similarity

The similarity measure comparison revealed that neural similarity was the strongest predictor of communicative success (BF = 6.16), indicating moderate evidence (Supplementary Figure S4; Supplementary Table S4). Relative comparison with the covariates of no interest model (BF = 1.88) indicates that the addition of neural similarity is three times as likely to predict communicative success (BF = 3.28).

#### Social cognitive × similarity interaction

**Fig. 1.**
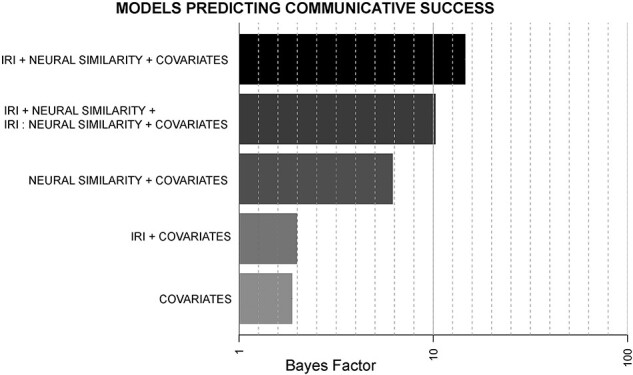
Main effects and interaction models predicting communicative success. The joint main effects model (IRI + neural similarity) and interaction term model (IRI × neural similarity) compared to single main effects and covariate-only models. Confederate, number of runs and motion similarity were included in all models. *Note:* The *x*-axis is a log scale, not linear.

**Fig. 2.**
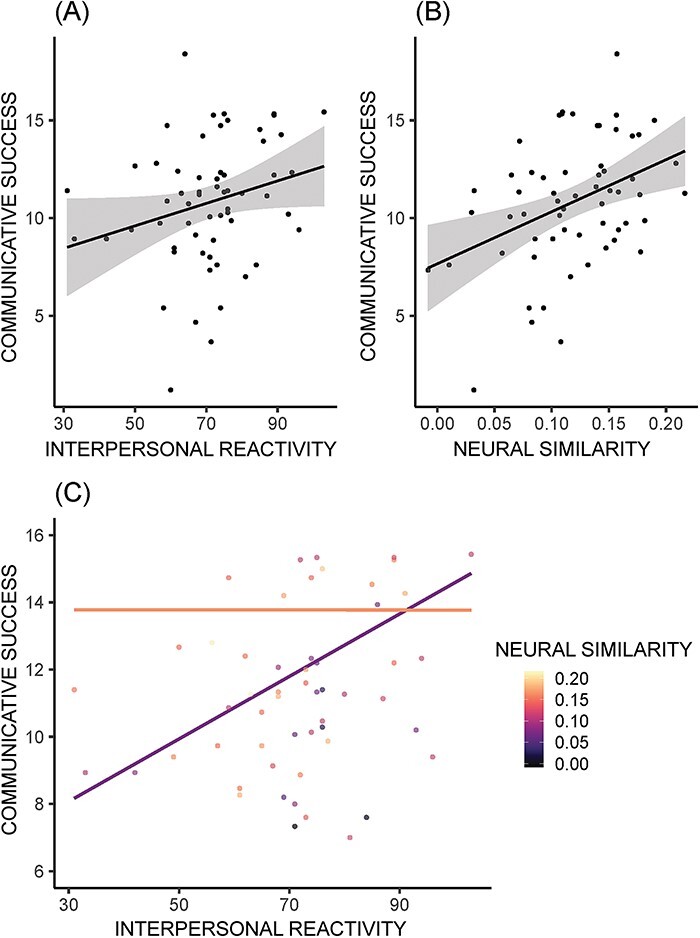
Associations between predictive measures and communicative success. Average distance scores were inverted prior to plotting, so higher scores indicate greater success. (A) IRI scores plotted against communicative success. (B) Neural similarity to partner plotted against communicative success. (C) Regression lines for the association between IRI and communicative success with the interactive effect of neural similarity. Hotter colors indicate higher neural similarity, and plot lines are ±1 standard deviation (SD) from mean neural similarity. All models include confederate as a covariate, and the models with neural similarity include the number of runs and motion similarity as a covariate.

IRI and neural similarity were entered into a subsequent interaction model comparison test. This test revealed strong evidence (BF > 10) for both main effects and the interaction between them (Figure 1; Table 2). The pattern of results was as expected, where both higher similarity and higher IRI scores predicted communicative success (Figure 2A,B), and the relationship between IRI and success was stronger when similarity was low (Figure 2C). The additive model where each main effect contributed separately to communicative success proved to be slightly stronger than the interaction model, with a relative BF of the separate additive model slightly greater than that of the model including the interaction term (BF_maineffects_/BF_interaction_ = 1.43). Both the additive and the interaction term models also proved to be more than three times likely to predict communicative success than the effect of the covariates alone.

**Table 2.** Main effects and interaction models predicting communicative success. Bayes Factors (BF) and relative BFs (compared to the covariates alone) for all possible combinations of the two predictors: interpersonal reactivity index (IRI) scores and neural similarity to partner. Covariates include: Confederate partner, number of runs of neural similarity data, and motion similarity (framewise displacement correlation to partner)

Model	BF	BF_model_/BF_covariates_
IRI + neural similarity + covariates	14.54	7.77
IRI + neural similarity + IRI:neural similarity + covariates	10.21	5.46
Neural similarity + covariates	6.17	3.30
IRI + covariates	1.97	1.05
Covariates	1.87	1

scFDwith the

#### Exploratory region of interest analysis

**Fig. 3.**
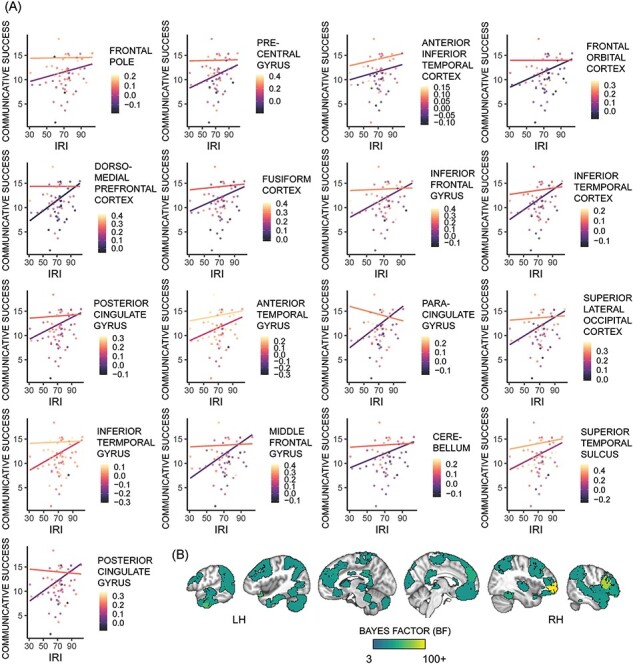
Brain regions predicting communicative success in the joint main effects model. Average distance scores were inverted prior to plotting, so higher scores indicate greater success. (A) Regression lines for models which yielded a BF ≥ 10 when neural similarity within the region was included. Hotter colors indicate higher neural similarity, and plot lines are ±1SD from mean neural similarity. All models include confederate, number of runs and motion similarity as covariates. (B) Map of regions where the joint main effects model yielded a BF > 3 when neural similarity within the region was included.

To examine which brain regions might be contributing to these results, we ran the winning model (Communicative Success = Neural Similarity + IRI + Confederate + Number of Runs + FD Similarity) using individual region data from the Shen atlas. Figure 3 shows the resulting regions with moderate or strong evidence. These regions spanned several functional networks defined by Finn and Shen and colleagues (Shen *et al.*, 2013; Finn *et al.*, 2015), including the medial frontal, frontoparietal, visual association and default mode. The list of all BF > 3 regions and associated functional networks are given in Supplementary Table S14.

### Perceived interaction quality

#### Social cognitive ability

**Fig. 5.**
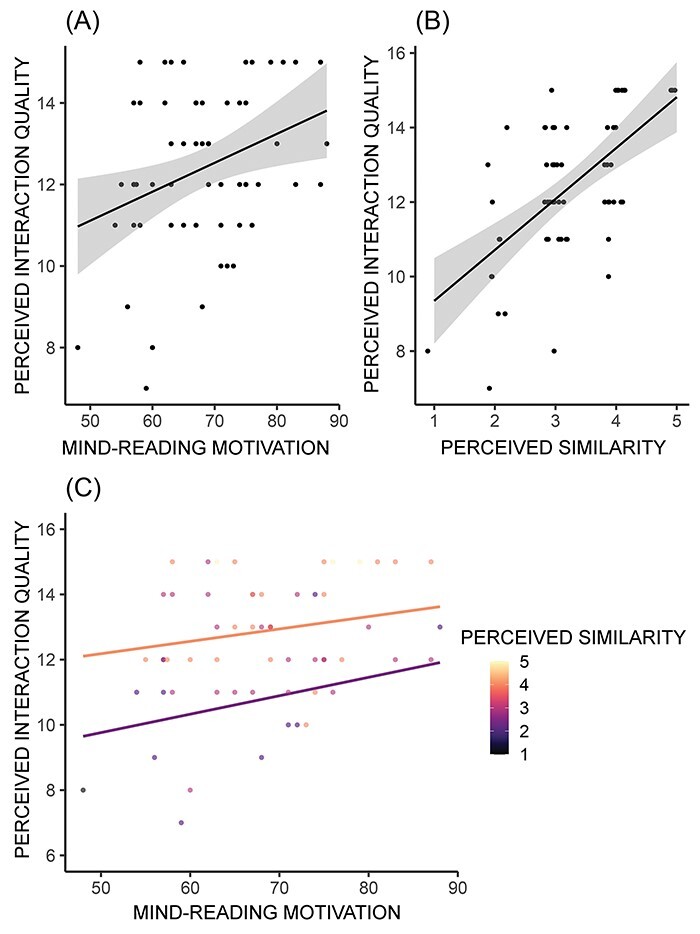
Associations between predictive measures and perceived interaction quality. (A) MRMS scores plotted against perceived interaction quality. (B) Perceived similarity to partner plotted against perceived interaction quality. Points are jittered randomly by 0.2 units on the *x*-axis. (C) Regression lines for the association between MRMS and perceived interaction quality with the effect of perceived similarity. Hotter colors indicate higher perceived similarity, and plot lines are ±1SD from mean perceived similarity. All models include confederate as a covariate.

The social cognitive measure comparison (*n* = 58) revealed that no measures yielded more than anecdotal evidence against the null in predicting perceived interaction quality (Supplementary Figure S10; Supplementary Table S10). Total score on the MRMS was the strongest predictor of perceived interaction quality (BF = 2.15; Figure 5A), so it was chosen for the follow-up interaction analysis.

#### Similarity

The similarity measure comparison revealed that self-reported perceived similarity was the most predictive measure of perceived interaction quality (BF = 257.99; Figure 5B), indicating very strong evidence (Supplementary Figure S11; Supplementary Table S11) and even stronger evidence when compared to the covariate-only model (BF = 9932.30). Unlike communicative success, neural similarity did not predict perceived interaction quality (BF = 0.02).

#### Social cognitive ×similarity interaction

**Fig. 4.**
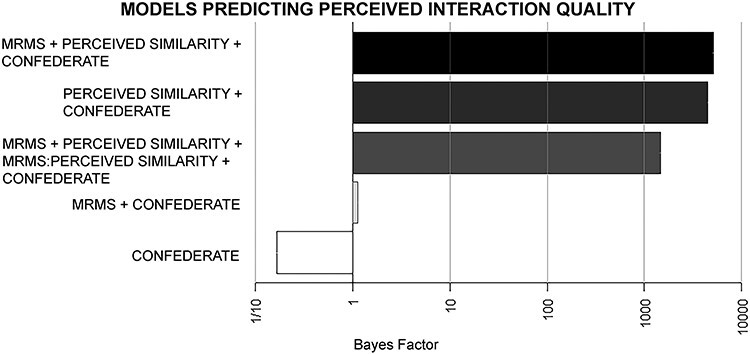
Main effects and interaction models predicting perceived interaction quality. The joint main effects model (MRMS + perceived similarity), interaction term model (MRMS × perceived similarity) and single main effects models compared to the confederate only model. *Note:* The *x*-axis is a log scale, not linear.

MRMS and perceived similarity were entered into a subsequent interaction model comparison test. Similar to cooperative success, the model which best predicted perceived interaction quality was the separate contribution of both main effects without the interaction, although the addition of MRMS scores did not markedly improve the model (BF_maineffects_/BF_perceivedsimilarity_ = 1.13). All three models where perceived similarity was included showed extremely strong evidence toward predicting perceived interaction quality (BF > 100), which was much stronger than the contribution of confederate alone (Figure 4; Table 3). The direction of results was as expected, where greater perceptions of similarity are strongly associated with greater perceptions of interaction quality, with a much weaker positive relationship between mind-reading motivation and perceived interaction quality (Figure 5).

**Table 3.** Main effects and interaction models predicting perceived interaction qualityBayes Factors (BF) and relative BFs (compared to the covariates alone) for all possible combinations of the two predictors: mind-reading motivation (MRMS) scores and perceived similarity to partner. The effect of confederate was included in all models

Model	BF	BF_model_/BF_confederate_
MRMS + perceived similarity + confederate	5070.88	30285.92
Perceived similarity + confederate	4445.42	26550.32
MRMS + perceived similarity + MRMS:perceived similarity + confederate	1452.87	8677.27
MRMS + confederate	1.13	6.75
Confederate	0.17	1.00

## Discussion

This study examined the individual and dyadic predictors that support social interaction quality between strangers. We found that trait empathy as well as neural similarity to an interaction partner is positively associated with communicative success with that partner, revealing the importance of sharing perspectives in communication. On the other hand, perceived similarity to partner is associated with perceived interaction quality, showing that neurobiological similarity is not as important as assumed similarity when promoting positive feelings about an interaction.

### Sharing perspectives predicts communicative success

Communicative success with an unfamiliar partner was most strongly predicted by the joint contributions of neural similarity to that partner and individual trait global empathy assessed by the IRI. Both yielded positive relationships: greater neural similarity and greater total IRI scores were associated with greater communicative success. These results indicate that it is important to share perspectives in order to successfully communicate: higher empathy indicates a greater propensity to put oneself into the shoes of a partner (Davis, 1980), and higher neural similarity indicates that one already experiences the world similar to that partner. Generalized shared reality has been defined as the subjective experience of sharing a set of inner states (e.g. thoughts, feelings or beliefs) in common with an interaction partner, and theory suggests that this shared reality enhances both interpersonal connection and a sense of understanding about the world. New acquaintances might discuss a number of specific topics to establish common ground, but, through this process, dyads create a more general shared information space spanning multiple domains (Stolk *et al.*, 2014; Rossignac-Milon and Higgins, 2018; Rossignac-Milon *et al.*, 2021). Our results suggest that this is more successful among those who have higher empathic traits and those who already share similar ways of processing the world with their partner. These initial successful communication endeavors among strangers may go on to support forming healthy relationships, as both empathy and neural similarity have been linked to closer and more supportive friendships (Hruschka, 2010; Ciarrochi *et al.*, 2017; Parkinson *et al.*, 2018; Hyon *et al.*, 2020). Although the separate contributions of empathy and neural similarity were found to be the strongest predictors, we also found evidence for an interaction between them: among pairs with low neural similarity, communication success was preserved if participants had higher global empathy. This suggests that individual social-emotional traits can, in some cases, make up for dissimilarity among people who are trying to understand each other. In contrast, among those who begin from a more similar perspective, the ability to share perspectives is not necessary for communication success.

Exploratory analyses revealed that a number of networks apart from primary sensory regions were driving the neural similarity effect in predicting communicative success. These included medial frontal, frontoparietal, default mode, subcortical-cerebellum and visual association networks. Similarity in the default mode network is implicated in a variety of cognitive processes and considered a ‘sense-making’ hub connecting the internal self to external context (Yeshurun *et al.*, 2021). Some of these regions (e.g. lateral occipital cortex, temporoparietal junction, inferior frontal gyrus, precuneus and both dorsomedial and ventromedial prefrontal cortices) have shown greater similarity among people who share interpretations or perspectives of events when context was experimentally manipulated (Lahnakoski *et al.*, 2014; Yeshurun *et al.*, 2017). Furthermore, similarity in some regions (e.g. dorsal striatum, involved in narrative memory (Ben-Yakov and Dudai, 2011); posterior parietal cortex, involved in selective attention (Behrmann *et al.*, 2004)) have been linked to friendship in the real world (Parkinson *et al.*, 2018). Our data were not collected during a task, so specific cognitive processes can only be speculated, but results suggest a link between these outcomes from prior work. Among strangers, greater neural similarity in regions which reflect similar psychological perspectives is associated with greater communicative success during a social interaction. This success may then support burgeoning real-world friendships.

### Perceived similarity predicts subjective interaction quality

In contrast to communicative success, perceived interaction quality was predicted most strongly by perceived similarity and not by neural similarity. These results indicate that feeling similar to a partner is in some cases more important than actually being similar. These feelings of similarity may be an important marker of belongingness and inclusion. Interestingly, the two similarity measures were uncorrelated with each other (*r* = −0.17, n.s.; Supplementary Figure S2). The interaction was relatively brief, meaning the amount of information that participants were able to share with each other was limited in scope, so it is possible that some felt more similar to their partner simply because they found common ground quickly (e.g. two partners who share the same major and end up discussing commonalities around school, rather than two partners who both love surfing, but the topic was never raised). Further work with larger samples and stronger friendships should be done to confirm this relationship, or lack thereof, between neural and perceptual similarity. Even so, our results suggest that perceiving similarities with another is a strong enough signal to promote positive feelings about an interaction, independent of communicative success on a task. This could have implications for solving conflicts and bridging divides; if feelings of similarity can be induced, then interactions among dissimilar people may prove to be more successful. A large field of work has examined abstract or minimal group similarities, which have negative societal consequences, particularly for those considered dissimilar (e.g. stereotypes, prejudice and conflict; Hewstone *et al.*, 2002; Dunham, 2018). However, our results align with more prosocial consequences of perceived similarity, such as increasing desire to form friendships (Burger *et al.*, 2004; Guéguen *et al.*, 2011; Martin *et al.*, 2013). If these perceptions are the important drivers of perceived interaction quality, then biological similarity may not be needed to establish common ground and engage in healthy and positive interactions.

This distinction between neural similarity and perceived similarity may be relevant in understanding communicative success in populations that exhibit social interaction differences as well. For example, similarity in autistic traits or diagnosis, rather than social ability, is associated with greater self-reported friendship quality and greater interpersonal rapport (Crompton *et al.*, 2020; Bolis *et al.*, 2021). However, fMRI studies show more idiosyncratic neural patterns compared to typically developing groups and among autistic individuals (Hasson *et al.*, 2009; Salmi *et al.*, 2013; Byrge *et al.*, 2015; Bolton *et al.*, 2018; Lyons *et al.*, 2020). It will be important to compare whether neural similarity or perceived similarity contributes more significantly to successful social interaction and feelings of belonging among a wider range of individuals.

### Social cognitive ability does not predict subjective interaction quality

The addition of mind-reading motivation scores to perceived similarity modestly improved upon the model predicting perceived interaction quality, but mind-reading motivation was not a good predictor of perceived quality on its own. In fact, no individual trait or task social cognitive measures reached a BF commensurate with moderate evidence against the null hypothesis. This is consistent with previous work which has not shown a positive association between theory of mind ability and self- or partner-generated ratings of interaction quality (Alkire *et al.*, 2023). It is likely that casual introductory social interactions do not require good mind-reading skills in order to be successful, especially if the success measure is subjective quality rather than task-based performance, which more overtly requires communicative understanding.

## Limitations

This study had several limitations. First, we trained confederate research assistants to be partners rather than pairing naïve participants together. This maximized efficiency in data collection, allowing for a greater number of participants to be scanned without halving the number of similarity data points, but it also reduced the variability of neural responses with which to compare participant neural activity. Additionally, we controlled for verbal information content that participants received across the communicative game as we did not want the quality of the clue from the clue giver (i.e. how ‘good’ or ‘bad’ a clue was in explaining the bullseye location) to determine the success of the guesser; however, this introduced a level of artificiality into the task, which may explain differences observed between perceived and neural similarity. Even with identical clues, however, we found that neural similarity to their partner predicted success in determining bullseye location, suggesting that they were using more than simple verbal signals. Finally, to maximize the possibility that differences in the results are due to individual (or dyadic) differences, we did not counterbalance any of the task trials or naturalistic videos participants viewed in the scanner. However, this fixed order may limit the generalizability of relative contributions of each measure. Relatedly, the outcome measures were based on different portions of the interaction, which made comparisons between them not perfectly matched. Future work could incorporate a collaborative task during conversation and limit ratings on interaction quality just to that interaction.

## Conclusions

This study examined the individual and dyadic factors that support successful social interactions with strangers. Our results reveal that both neural similarity and empathy are associated with successful communication. Additionally, perceived similarity strongly supports feelings of interaction quality, showing that neural and perceived similarities are distinctly involved in different types of interaction success. These results indicate the importance of sharing perspectives with one’s partner for communicating and suggest that even independent of neurobiological similarity and communication success, positive feelings about social interactions can be gained with perceived similarity between social partners.

## Supplementary Material

nsad045_Supp

## Data Availability

The data from consenting participants that support the findings of this study have been uploaded to the National Institute of Mental Health Data Archive (NDA) under collection #2661 and are available upon request at https://nda.nih.gov. This project was pre-registered, and supplementary materials was added to the Open Science Framework at https://osf.io/f3nu7.

## References

[R1] Alkire D. , McNaughton K.A., Yarger H.A., Shariq D., Redcay E. (2023). Theory of mind in naturalistic conversations between autistic and typically developing children and adolescents. *Autism*, 27(2), 472–88. 35722978 10.1177/13623613221103699 PMC9763550

[R2] Baumeister R.F. , Leary M.R. (1995). The need to belong: desire for interpersonal attachments as a fundamental human motivation. *Psychological Bulletin*, 117(3), 497–529. 7777651

[R3] Behrmann M. , Geng J.J., Shomstein S. (2004). Parietal cortex and attention. *Current Opinion in Neurobiology*, 14(2), 212–7. 15082327 10.1016/j.conb.2004.03.012

[R4] Ben-Yakov A. , Dudai Y. (2011). Constructing realistic engrams: poststimulus activity of hippocampus and dorsal striatum predicts subsequent episodic memory. *Journal of Neuroscience*, 31(24), 9032–42. 21677186 10.1523/JNEUROSCI.0702-11.2011 PMC6622928

[R5] Bernieri F.J. Rosenthal R. (1991). Interpersonal coordination: behavior matching and interactional synchrony. In: Feldman, R.S., Rime, B., editors. *Fundamentals of Nonverbal Behavior*. Cambridge, UK: Cambridge University Press, 401–32.

[R6] Blanke E.S. , Riediger M. (2019). Reading thoughts and feelings in other people: empathic accuracy across adulthood. *Progress in Brain Research*, 247, 305–27. 31196439 10.1016/bs.pbr.2019.02.002

[R7] Bolis D. , Lahnakoski J.M., Seidel D., Tamm J., Schilbach L. (2021). Interpersonal similarity of autistic traits predicts friendship quality. *Social Cognitive and Affective Neuroscience*, 16(1-2), 222–31. 33104781 10.1093/scan/nsaa147 PMC7812635

[R8] Bolton T.A.W. , Jochaut D., Giraud A., Van De Ville D. (2018). Brain dynamics in ASD during movie‐watching show idiosyncratic functional integration and segregation. *Human Brain Mapping*, 39(6), 2391–404. 29504186 10.1002/hbm.24009 PMC5969252

[R9] Brown-Schmidt S. Heller D. (2018) Perspective-taking during conversation. In: Gaskell, G., Rueschemeyer, S.A., editors. *Oxford Handbook of Psycholinguistics*. Oxford, UK: Oxford University Press, 549–72.

[R10] Burger J.M. , Messian N., Patel S., Del Prado A., Anderson C. (2004). What a coincidence! The effects of incidental similarity on compliance. *Personality & Social Psychology Bulletin*, 30(1), 35–43. 15030641 10.1177/0146167203258838

[R11] Byrge L. , Dubois J., Tyszka J.M., Adolphs R., Kennedy D.P. (2015). Idiosyncratic brain activation patterns are associated with poor social comprehension in autism. *Journal of Neuroscience*, 35(14), 5837–50. 25855192 10.1523/JNEUROSCI.5182-14.2015 PMC4388936

[R12] Carpenter J.M. , Green M.C., Vacharkulksemsuk T. (2016). Beyond perspective-taking: mind-reading motivation. *Motivation and Emotion*, 40(3), 358–74.

[R13] Ciarrochi J. , Parker P.D., Sahdra B.K., Kashdan T.B., Kiuru N., Conigrave J. (2017). When empathy matters: the role of sex and empathy in close friendships. *Journal of Personality*, 85(4), 494–504. 27012715 10.1111/jopy.12255

[R14] Crompton C.J. , Sharp M., Axbey H., Fletcher-Watson S., Flynn E.G., Ropar D. (2020). Neurotype-matching, but not being autistic, influences self and observer ratings of interpersonal rapport. *Frontiers in Psychology*, 11, 586171. 10.3389/fpsyg.2020.586171 PMC7645034 33192918

[R15] Cui X. , Bryant D.M., Reiss A.L. (2012). NIRS-based hyperscanning reveals increased interpersonal coherence in superior frontal cortex during cooperation. *NeuroImage*, 59(3), 2430–7. 21933717 10.1016/j.neuroimage.2011.09.003 PMC3254802

[R16] Davis M.H. (1980). A multidimensional approach to individual differences in empathy. *JSAS Catalog of Selected Documents in Psychology*, 10, 85.

[R17] Davis M.H. (1983). Measuring individual differences in empathy: evidence for a multidimensional approach. *Journal of Personality and Social Psychology*, 44(1), 113–26.

[R18] Depue R.A. , Morrone-Strupinsky J.V. (2005). A neurobehavioral model of affiliative bonding: implications for conceptualizing a human trait of affiliation. *Behavioral and Brain Sciences*, 28(03), 313–49. 16209725 10.1017/S0140525X05000063

[R19] Djalovski A. , Dumas G., Kinreich S., Feldman R. (2021). Human attachments shape interbrain synchrony toward efficient performance of social goals. *NeuroImage*, 226, 117600. 10.1016/j.neuroimage.2020.117600 33249213

[R20] Dumas G. , Nadel J., Soussignan R., Martinerie J., Garnero L., Lauwereyns J. (2010). Inter-brain synchronization during social interaction. *PLoS One*, 5(8), e12166. 10.1371/journal.pone.0012166 PMC2923151 20808907

[R21] Dunham Y. (2018). Mere membership. *Trends in Cognitive Sciences*, 22(9), 780–93. 30119749 10.1016/j.tics.2018.06.004

[R22] Epley N. , Schroeder J. (2014). Mistakenly seeking solitude. *Journal of Experimental Psychology. General*, 143(5), 1980–99. 25019381 10.1037/a0037323

[R23] Esteban O. , Markiewicz C.J., Blair R.W., et al. (2018). fMRIPrep: a robust preprocessing pipeline for functional MRI. *Nature Methods*, 16, 111–6. 30532080 10.1038/s41592-018-0235-4 PMC6319393

[R24] Finn E.S. , Shen X., Scheinost D., et al. (2015). Functional connectome fingerprinting: identifying individuals using patterns of brain connectivity. *Nature Neuroscience*, 18(11), 1664–71. 26457551 10.1038/nn.4135 PMC5008686

[R25] Frith C.D. , Frith U. (2006). The neural basis of mentalizing. *Neuron*, 50(4), 531–4. 16701204 10.1016/j.neuron.2006.05.001

[R26] Geen R.G. (1991). Social motivation. *Annual Review of Psychology*, 42(1), 377–99. 10.1146/annurev.ps.42.020191.002113 2018398

[R27] Geuter S. , Qi G., Welsh R.C., Wager T.D., Lindquist M.A. (2018). Effect size and power in fMRI group analysis [Preprint]. *bioRxiv*, 295048.

[R28] Gleason K.A. , Jensen-Campbell L.A., Ickes W. (2009). The role of empathic accuracy in adolescents’ peer relations and adjustment. *Personality & Social Psychology Bulletin*, 35(8), 997–1011. 19498068 10.1177/0146167209336605

[R29] Golan O. , Baron-Cohen S., Hill J. (2006). The Cambridge Mindreading (CAM) face-voice battery: testing complex emotion recognition in adults with and without Asperger Syndrome. *Journal of Autism and Developmental Disorders*, 36(2), 169–83. 16477515 10.1007/s10803-005-0057-y

[R30] Granovetter M.S. (1973). The strength of weak ties. *American Journal of Sociology*, 78(6), 1360–80.

[R31] Guéguen N. , Martin A., Meineri S. (2011). Similarity and social interaction: when similarity fosters implicit behavior toward a stranger. *The Journal of Social Psychology*, 151(6), 671–3. 22208106 10.1080/00224545.2010.522627

[R32] Hasson U. , Avidan G., Gelbard H., et al. (2009). Shared and idiosyncratic cortical activation patterns in autism revealed under continuous real-life viewing conditions. *Autism Research*, 2(4), 220–31. 19708061 10.1002/aur.89 PMC2775929

[R33] Hewstone M. , Rubin M., Willis H. (2002). Intergroup bias. *Annual Review of Psychology*, 53(1), 575–604. 10.1146/annurev.psych.53.100901.135109 11752497

[R34] Holper L. , Scholkmann F., Wolf M. (2012). Between-brain connectivity during imitation measured by fNIRS. *NeuroImage*, 63(1), 212–22. 22732563 10.1016/j.neuroimage.2012.06.028

[R35] Holt-Lunstad J. (2018). Why social relationships are important for physical health: a systems approach to understanding and modifying risk and protection. *Annual Review of Psychology*, 69(1), 437–58. 10.1146/annurev-psych-122216-011902 29035688

[R36] Holt-Lunstad J. , Smith T.B., Layton J.B., Brayne C. (2010). Social relationships and mortality risk: a meta-analytic review. *PLoS Medicine*, 7(7), e1000316. 10.1371/journal.pmed.1000316 PMC2910600 20668659

[R37] Hruschka D.J. (2010). The development of friendships. In: *Friendship: Development, Ecology, and Evolution of a Relationship*. Berkeley, CA: University of California Press, 146–67.

[R38] Hyon R. , Youm Y., Kim J., Chey J., Kwak S., Parkinson C. (2020). Similarity in functional brain connectivity at rest predicts interpersonal closeness in the social network of an entire village. *Proceedings of the National Academy of Sciences*, 117(52), 33149–60. 10.1073/pnas.2013606117 PMC7777022 33318188

[R39] Jiang J. , Dai B., Peng D., Zhu C., Liu L., Lu C. (2012). Neural synchronization during face-to-face communication. *Journal of Neuroscience*, 32(45), 16064–9. 23136442 10.1523/JNEUROSCI.2926-12.2012 PMC6621612

[R40] Jiang J. , Zheng L., Lu C. (2021). A hierarchical model for interpersonal verbal communication. *Social Cognitive and Affective Neuroscience*, 16(1–2), 246–55. 33150951 10.1093/scan/nsaa151 PMC7812628

[R41] Kingsbury L. , Hong W. (2020). A multi-brain framework for social interaction. *Trends in Neurosciences*, 43(9), 651–66. 32709376 10.1016/j.tins.2020.06.008 PMC7484406

[R42] Kinreich S. , Djalovski A., Kraus L., Louzoun Y., Feldman R. (2017). Brain-to-brain synchrony during naturalistic social interactions. *Scientific Reports*, 7(1), 17060. 10.1038/s41598-017-17339-5 PMC5719019 29213107

[R43] Kruschke J.K. , Liddell T.M. (2018). The Bayesian New Statistics: hypothesis testing, estimation, meta-analysis, and power analysis from a Bayesian perspective. *Psychonomic Bulletin & Review*, 25(1), 178–206. 28176294 10.3758/s13423-016-1221-4

[R44] Kuiper J.S. , Zuidersma M., Oude Voshaar R.C., et al. (2015). Social relationships and risk of dementia: a systematic review and meta-analysis of longitudinal cohort studies. *Ageing Research Reviews*, 22, 39–57. 25956016 10.1016/j.arr.2015.04.006

[R45] Lahnakoski J.M. , Glerean E., Jääskeläinen I.P., et al. (2014). Synchronous brain activity across individuals underlies shared psychological perspectives. *NeuroImage*, 100, 316–24. 24936687 10.1016/j.neuroimage.2014.06.022 PMC4153812

[R46] Lecce S. , Ceccato I., Bianco F., Rosi A., Bottiroli S., Cavallini E. (2017). Theory of mind and social relationships in older adults: the role of social motivation. *Aging & Mental Health*, 21(3), 253–8. 26581839 10.1080/13607863.2015.1114586

[R47] Lee M.D. , Wagenmakers E.J. (2013). *Bayesian Cognitive Modeling: A Practical Course*. Cambridge: Cambridge University Press.

[R48] Liang F. , Paulo R., Molina G., Clyde M.A., Berger J.O. (2008). Mixtures of *g* priors for Bayesian variable selection. *Journal of the American Statistical Association*, 103(481), 410–23.

[R49] Lyons K.M. , Stevenson R.A., Owen A.M., Stojanoski B. (2020). Examining the relationship between measures of autistic traits and neural synchrony during movies in children with and without autism. *NeuroImage: Clinical*, 28, 102477. 10.1016/j.nicl.2020.102477 PMC7680702 33395970

[R50] Martin A. , Jacob C., Guéguen N. (2013). Similarity facilitates relationships on social networks: a field experiment on Facebook. *Psychological Reports*, 113(1), 217–20. 10.2466/21.07.pr0.113x15z8 24340812

[R51] Mauersberger H. , Tune J.L., Kastendieck T., Czarna A.Z., Hess U. (2022). Higher heart rate variability predicts better affective interaction quality in non‐intimate social interactions. *Psychophysiology*, 59(11), e14084. 10.1111/psyp.14084 35569090

[R52] McManus I.C. , Jonvik H., Richards P., Paice E. (2011). Vocation and avocation: leisure activities correlate with professional engagement, but not burnout, in a cross-sectional survey of UK doctors. *BMC Medicine*, 9(1), 100. 10.1186/1741-7015-9-100 PMC3196901 21878123

[R53] Morey R.D. , and Rouder J.N. (2018). BayesFactor: computation of Bayes factors for common designs (R package version 0.9.12-4.2).

[R54] Mote J. , Gard D.E., Gonzalez R., Fulford D., Lincoln S.H. (2019). How did that interaction make you feel? The relationship between quality of everyday social experiences and emotion in people with and without schizophrenia. *PLoS One*, 14(9), e0223003. 10.1371/journal.pone.0223003 PMC6768461 31568483

[R55] Nadig A. , Seth S., Sasson M. (2015). Global similarities and multifaceted differences in the production of partner-specific referential pacts by adults with autism spectrum disorders. *Frontiers in Psychology*, 6, 1888. 10.3389/fpsyg.2015.01888 PMC4681838 26733897

[R56] Nastase S.A. , Gazzola V., Hasson U., Keysers C. (2019). Measuring shared responses across subjects using intersubject correlation. *Social Cognitive and Affective Neuroscience*, 14(6), 667–85. 31099394 10.1093/scan/nsz037 PMC6688448

[R57] Nguyen T. , Schleihauf H., Kayhan E., Matthes D., Vrtička P., Hoehl S. (2020). The effects of interaction quality on neural synchrony during mother-child problem solving. *Cortex*, 124, 235–49. 31927470 10.1016/j.cortex.2019.11.020

[R58] Nilsen E.S. , Bacso S.A. (2017). Cognitive and behavioural predictors of adolescents’ communicative perspective‐taking and social relationships. *Journal of Adolescence*, 56(1), 52–63. 28157666 10.1016/j.adolescence.2017.01.004

[R59] Ong D.C. , Wu Z., Zhi-Xuan T., et al. (2021). Modeling emotion in complex stories: The Stanford Emotional Narratives Dataset. *IEEE Transactions on Affective Computing*, 12(3), 579–94. 34484569 10.1109/taffc.2019.2955949 PMC8414991

[R60] Palumbo R.V. , Marraccini M.E., Weyandt L.L., et al. (2017). Interpersonal autonomic physiology: a systematic review of the literature. *Personality and Social Psychology Review*, 21(2), 99–141. 26921410 10.1177/1088868316628405

[R61] Pan Y. , Cheng X., Zhang Z., Li X., Hu Y. (2017). Cooperation in lovers: an fNIRS-based hyperscanning study. *Human Brain Mapping*, 38(2), 831–41. 27699945 10.1002/hbm.23421 PMC6867051

[R62] Parkinson C. , Kleinbaum A.M., Wheatley T. (2018). Similar neural responses predict friendship. *Nature Communications*, 9(1), 332. 10.1038/s41467-017-02722-7 PMC5790806 29382820

[R63] Redcay E. , Schilbach L. (2019). Using second-person neuroscience to elucidate the mechanisms of social interaction. *Nature Reviews Neuroscience*, 20(8), 495–505. 31138910 10.1038/s41583-019-0179-4 PMC6997943

[R64] Reindl V. , Gerloff C., Scharke W., Konrad K. (2018). Brain-to-brain synchrony in parent-child dyads and the relationship with emotion regulation revealed by fNIRS-based hyperscanning. *NeuroImage*, 178, 493–502. 29807152 10.1016/j.neuroimage.2018.05.060

[R65] Rossignac-Milon M. , Bolger N., Zee K.S., Boothby E.J., Higgins E.T. (2021). Merged minds: generalized shared reality in dyadic relationships. *Journal of Personality and Social Psychology*, 120(4), 882–911. 32673045 10.1037/pspi0000266

[R66] Rossignac-Milon M. , Higgins E.T. (2018). Epistemic companions: shared reality development in close relationships. *Current Opinion in Psychology*, 23, 66–71. 29360060 10.1016/j.copsyc.2018.01.001

[R67] Rouder J.N. , Morey R.D. (2012). Default Bayes factors for model selection in regression. *Multivariate Behavioral Research*, 47, 877–903. 26735007 10.1080/00273171.2012.734737

[R68] Salmi J. , Roine U., Glerean E., et al. (2013). The brains of high functioning autistic individuals do not synchronize with those of others. *NeuroImage: Clinical*, 3, 489–97. 24273731 10.1016/j.nicl.2013.10.011 PMC3830058

[R69] Sandstrom G.M. , Dunn E.W. (2014). Social interactions and well-being: The surprising power of weak ties. *Personality & Social Psychology Bulletin*, 40(7), 910–22. 24769739 10.1177/0146167214529799

[R70] Saucier G. (1994). Mini-Markers: a brief version of Goldberg’s unipolar big-five markers. *Journal of Personality Assessment*, 63(3), 506–16. 7844738 10.1207/s15327752jpa6303_8

[R71] Savitsky K. , Keysar B., Epley N., Carter T., Swanson A. (2011). The closeness-communication bias: increased egocentrism among friends versus strangers. *Journal of Experimental Social Psychology*, 47(1), 269–73.

[R72] Schoot L. , Hagoort P., Segaert K. (2016). What can we learn from a two-brain approach to verbal interaction? *Neuroscience and Biobehavioral Reviews*, 68, 454–9. 27311632 10.1016/j.neubiorev.2016.06.009

[R73] Sedikides C. , Campbell W.K., Reeder G.D., Elliot A.J. (2002). The self in relationships: whether, how, and when close others put the self “in its place. *European Review of Social Psychology*, 12(1), 237–65.

[R74] Sened H. , Lavidor M., Lazarus G., Bar-Kalifa E., Rafaeli E., Ickes W. (2017). Empathic accuracy and relationship satisfaction: a meta-analytic review. *Journal of Family Psychology*, 31(6), 742–52. 28394141 10.1037/fam0000320

[R75] Seyfarth R.M. , Cheney D.L. (2012). The evolutionary origins of friendship. *Annual Review of Psychology*, 63(1), 153–77. 10.1146/annurev-psych-120710-100337 21740224

[R76] Shen X. , Tokoglu F., Papademetris X., Constable R.T. (2013). Groupwise whole-brain parcellation from resting-state fMRI data for network node identification. *NeuroImage*, 82, 403–15. 23747961 10.1016/j.neuroimage.2013.05.081 PMC3759540

[R77] Shockley K. , Richardson D.C., Dale R. (2009). Conversation and coordinative structures. *Topics in Cognitive Science*, 1(2), 305–19. 25164935 10.1111/j.1756-8765.2009.01021.x

[R78] Stolk A. , Noordzij M.L., Verhagen L., et al. (2014). Cerebral coherence between communicators marks the emergence of meaning. *Proceedings of the National Academy of Sciences*, 111(51), 18183–8. 10.1073/pnas.1414886111 PMC4280639 25489093

[R79] Sun J. , Harris K., Vazire S. (2020). Is well-being associated with the quantity and quality of social interactions? *Journal of Personality and Social Psychology*, 119(6), 1478–96. 31647273 10.1037/pspp0000272

[R80] Thoits P.A. (2011). Mechanisms linking social ties and support to physical and mental health. *Journal of Health and Social Behavior*, 52(2), 145–61. 21673143 10.1177/0022146510395592

[R81] Toni I. Stolk A. (2019). Conceptual alignment as a neurocognitive mechanism for human communicative interactions. In: Hagoort, P., editor. *Human Language: From Genes and Brains to Behavior*. Cambridge, MA: MIT Press, 249–56.

[R82] Wadge H. , Brewer R., Bird G., Toni I., Stolk A. (2019). Communicative misalignment in autism spectrum disorder. *Cortex*, 115, 15–26. 30738998 10.1016/j.cortex.2019.01.003

[R83] Wheatley T. , Boncz A., Toni I., Stolk A. (2019). Beyond the isolated brain: the promise and challenge of interacting minds. *Neuron*, 103(2), 186–8. 31319048 10.1016/j.neuron.2019.05.009 PMC7789915

[R84] Yeshurun Y. , Nguyen M., Hasson U. (2021). The default mode network: where the idiosyncratic self meets the shared social world. *Nature Reviews Neuroscience*, 22(3), 181–92. 33483717 10.1038/s41583-020-00420-w PMC7959111

[R85] Yeshurun Y. , Swanson S., Simony E., et al. (2017). Same story, different story: the neural representation of interpretive frameworks. *Psychological Science*, 28(3), 307–19. 28099068 10.1177/0956797616682029 PMC5348256

[R86] Yun K. , Watanabe K., Shimojo S. (2012). Interpersonal body and neural synchronization as a marker of implicit social interaction. *Scientific Reports*, 2(1), 959. 10.1038/srep00959 PMC3518815 23233878

[R87] Zaki J. , Bolger N., Ochsner K. (2008). It takes two: The interpersonal nature of empathic accuracy. *Psychological Science*, 19(4), 399–404. 18399894 10.1111/j.1467-9280.2008.02099.x

[R88] Zoom Video Communications Inc . (2016). Security guide, Zoom Video Communications Inc. Available: https://d24cgw3uvb9a9h.cloudfront.net/static/81625/doc/Zoom-Security-White-Paper.pdf.

