# Supplementary material for: Partner similarity and social cognitive traits predict social interaction success among strangers
Source: Soc Cogn Affect Neurosci. 2023 Sep 12;18(1):nsad045. doi: 10.1093/scan/nsad045 (PMC10516339; doi:10.1093/scan/nsad045)
Supplement: nsad045_Supp [file nsad045_supp.zip › scan-23-017-File010.pdf]

Supplementary Information

Table of Contents

Supplementary Methods **4**
Behavioral Session Setup 4
Semi-structured Partner Interaction Tasks 4
1. Get To Know You Task 4
2. Top Five List Task 4
3. Discussion Questions 5
4. Telewave Communication Game 5
Social Cognitive Measures 6
1. Complex Emotion Recognition in Faces Task 6
2. Empathic Accuracy from Emotional Narratives Task 7
3. Interpersonal Reactivity Index 7
4. Mind-Reading Motivation Scale 8
Similarity Measures 8
1. Big Five Personality Trait Mini-Markers 8
2. Modified Avocation Activities Questionnaire 8
fMRI Measures 11
Supplementary Table 2. Naturalistic Videos 12
fMRI Preprocessing Details 12
fMRI Data Quality Motion Check 18
Measurement Comparison 20
Supplementary Results **20**
Supplementary Table 3. Social cognitive measures predicting communicative success. 20
Supplementary Figure 3. Social cognitive measures predicting communicative success. 21
Supplementary Table 4. Similarity measures predicting communicative success. 22
Supplementary Figure 4. Similarity measures predicting communicative success. 23
Supplementary Table 5. Social cognitive measures predicting communicative success (edited Telewave scores). 23
Supplementary Figure 5. Social cognitive measures predicting communicative success (edited Telewave scores). 24
Supplementary Table 6. Similarity measures predicting communicative success (edited Telewave scores) 25
Supplementary Figure 6. Similarity measures predicting communicative success (edited Telewave scores) 26

Supplementary Table 7. Main effects and interaction models predicting communicative success (edited Telewave scores). 27
Supplementary Figure 7. Main effects and interaction models predicting communicative success (edited Telewave scores). 27
Supplementary Table 8. Social cognitive measures predicting communicative success (edited Face Task). 27
Supplementary Figure 8. Social cognitive measures predicting communicative success (edited Face Task). 28
Supplementary Table 9. Similarity measures predicting communicative success (Weighted Neural Similarity). 29
Supplementary Figure 9. Similarity measures predicting communicative success (Weighted Neural Similarity). 30
Supplementary Table 10. Cognitive and Emotional Empathy (IRI) Subscales predicting communicative success. 31
Supplementary Figure 10. Cognitive and Emotional Empathy (IRI) Subscales predicting communicative success. 31
Supplementary Table 11. Main effects and interaction model predicting communicative success (Emotional Empathy IRI Subscale). 31
Supplementary Figure 11. Main effects and interaction model predicting communicative success (Emotional Empathy IRI Subscale). 32
Supplementary Table 12. Social cognitive measures predicting perceived interaction quality. 32
Supplementary Figure 12. Social cognitive measures predicting perceived interaction quality. 33
Supplementary Table 13. Similarity measures predicting perceived interaction quality. 34
Supplementary Figure 13. Similarity measures predicting perceived interaction quality. 35
Supplementary Table 14. Social cognitive measures predicting perceived interaction quality (edited Face Task). 35
Supplementary Figure 14. Social cognitive measures predicting perceived interaction quality (edited Face Task). 36
Supplementary Table 15. Similarity measures predicting perceived interaction quality (Weighted Neural Similarity). 37
Supplementary Figure 15. Similarity measures predicting perceived interaction quality (Weighted Neural Similarity). 38
Supplementary Table 16. Brain Regions Predicting Communicative Success. 38
Supplementary Table 17. Sensitivity analysis varying prior distributions: Social Cognitive Measures predicting Communicative success. 42
Supplementary Table 18. Sensitivity analysis varying prior distributions: Similarity Measures predicting Communicative Success. 43

Supplementary Table 19. Sensitivity analysis varying prior distributions: Social Cognitive Measures predicting Perceived Interaction Quality. 45

Supplementary Table 20. Sensitivity analysis varying prior distributions: Similarity Measures predicting Perceived Interaction Quality. 46

Supplementary Table 21. Exploratory Interaction Models: Perceived Interaction Quality .. 47

Supplementary References 56

Supplementary Methods

Behavioral Session Setup

Participants were instructed to sit in front of their computer screen with head and shoulders in view and adjusted their Zoom settings so their partner's camera filled their entire screen, so that the virtual setup simulated a face-to-face interaction. They blurred their background if able and removed any hats or face masks that would occlude their face, to ensure that nonverbal signals such as facial expressions and upper body movements could be easily seen. Partners were gender matched, with the exception of two nonbinary participants who signed up for sessions with female partners. Behavioral tasks were virtually shared through PsychoPy3 (v2021.2.0) (Peirce et al., 2019) from the session administrator's computer, with mouse and keyboard control ceded to the participant. Participants then completed the remaining survey measures. Two measures were used as trait-level assessments of social cognitive ability and propensity, and two measures were used to examine similarity to interaction partner.

Semi-structured Partner Interaction Tasks

1. Get To Know You Task

Five minutes. The session administrator turned off their screen and microphone and allowed the two partners to meet and get to know each other through unstructured conversation.

2. Top Five List Task

Five minutes. In this task, the partners were asked to pick a category (movies, television shows, books or musical artists) and they worked together to create a single list of the best five items in that category. The confederate partners were instructed to allow the participant to choose (e.g., "I'd be fine with any of these. What do you think?").

3. Discussion Questions

Ten minutes. This task allowed participants to engage with each other on a deeper level with a list of questions which partners asked each other and then answered themselves. Several of the questions provided opportunities for participants to learn specific things about their confederate partner (who gave similar responses to every participant) that could then be used in the interactive task that followed (i.e., telewave). The questions were as follows:

1. Describe your ideal day to your partner.
2. What did you want to be as a child?
3. What would your younger self not believe about your life today?
4. What are you passionate about?
5. Does your partner seem like more of a morning person or a night owl? Why?
6. Does your partner seem like more of a creative or analytical type? Why?
7. What is similar and what is different about you and your partner? Discuss.

4. Telewave Communication Game

Thirty minutes. This is an online cooperative "mindreading" game (https://github.com/gjeuken/telewave). In this game, one partner is designated as the **clue giver** and the other is the **guesser**. The clue giver receives a prompt on a binary scale (e.g., Hot-Cold) and they are shown a bulls-eye correct answer between 0-100 (which the guesser does not see). They must then give a clue to their partner to guide their partner to where the bulls-eye is located on the scale. For example, if the game board was "Cold-Hot" and the bulls-eye was slightly to the left of center, a clue might be something a little colder than room temperature such as "salad". The participant was designated as the clue giver and confederate as guesser first. After 15 rounds, the roles were reversed so the participant was the guesser (and confederate clue giver) for the final 15 rounds. Only the trials in which the participant was the guesser were analyzed since the confederate was familiar with all correct answers, making those scores invalid. They were told that the goal of the game was to work together to score as many bulls-eye points as possible. All confederates gave identical clues to the participants; these clues had been developed organically by confederates in their first playing of

the game. The exception was in cases when the clue would directly contradict something that they had discussed together during the first three tasks. In this case, the confederates were trained to give as similar as possible a clue (e.g., if the participant said they loved Ariana Grande, the confederate would not give that musical artist as an example of someone who was "overrated"; they might choose "Lady Gaga" instead). Sixteen subjects had one clue changed, three subjects had two clues changed, and three subjects had three clues changed. Success was operationalized as the distance between the participant's guess and the correct bulls-eye, averaged over all trials. Average distance was used instead of summed distance as two subject's first trials were removed due to user error (i.e., the participant accidentally peeked at the correct answer before guessing). Secondary analyses with the clues which deviated from the planned script removed from this average did not change the pattern of results (**Supplementary Results: Supplementary Tables 4-6; Supplementary Figures 5-7**). We selected this game as our measure of communicative success for the following reasons: the task was cooperative so as to promote a shared goal; engaging so that partners would have the potential to enjoy the interaction; and allowed for a simple way to compare results across pairs (i.e., trials resulted in a number describing how correct or incorrect they were). We had originally planned for partners to play a physical game of Tangrams, but when the pandemic forced campus closures and it was no longer safe to conduct in-person studies, we switched to a task that was easy to play virtually.

Social Cognitive Measures

1. Complex Emotion Recognition in Faces Task

(Golan et al., 2006). Subjects were shown brief (5s) videos of human faces displaying a variety of internal cognitive and affective states and were asked to choose which of four options most closely matched the video. They were first presented with the options and the corresponding number to press and were allowed to ask for definitions of any of the options, which were read to them from

a definition sheet by the session administrator. Once they pressed a button to continue, they saw the video above the options, which repeated once before moving on to the next trial. Performance was measured as percent accuracy across 50 trials and non-responses were treated as incorrect. One participant only completed 25 trials due to computer error. The subject was included as they were not an outlier, but secondary analyses with this subject removed also did not change the pattern of results (**Supplementary Results: Supplementary Table 7 & 11; Supplementary Figure 8 & 12**).

2. **Empathic Accuracy from Emotional Narratives Task**

(Ong et al., 2021). Subjects were shown four videos (average length = 2 minutes) of college students telling emotional personal narratives. They were asked to continuously rate the valence of the person in the video during the storytelling, using their mouse, on a sliding scale from Very Negative to Very Positive. Performance was measured as the correlation between participant ratings and ratings gathered from the narrator of their own emotional states after the video was recorded. Performance on one of the videos was consistently low (group average $r = -0.11$) and negatively correlated with performance on the other three videos ($r = -0.154$; $r = -0.126$; $r = -0.241$), so it was discarded. The average correlation across the remaining three videos was used as a single measure of empathic accuracy. Seven subjects did not provide any ratings for one other video so correlations to target could not be conducted; the average of the remaining two videos was used in these cases.

3. **Interpersonal Reactivity Index**

(Davis, 1980; 1983). This 28-item measure assesses the reactions of an individual to the observed experiences of others. It is comprised of four subscales of empathic constructs assessed on a 5-point Likert scale ranging from “Does not describe me well” to “Describes me very well”. The subscales are Perspective Taking (the tendency to spontaneously adopt the psychological point of view of others), Fantasy (the tendency to imagine feeling and acting as fictitious characters),

Empathic Concern (the tendency to feel sympathy and concern for unfortunate others), and Personal Distress (the tendency to experience "self-oriented" feelings of anxiety and unease in tense interpersonal settings). The sum total of all items was used as a measure of global empathy.

4. **Mind-Reading Motivation Scale**

(Carpenter et al., 2016). This 13-item measure assesses an individual's propensity toward engaging with others' perspectives and mental states on a 7-point Likert scale from "Strongly Disagree" to "Strongly Agree".

Similarity Measures

1. **Big Five Personality Trait Mini-Markers**

(Saucier, 1994). This 40-item measure is a robust subset of the original 100-item Big Five Personality factor structure (Goldberg, 1992), which examines personality characteristics on a 9-point Likert scale from "Extremely Inaccurate" to "Extremely Accurate". For this study, we were interested in similarity in personality traits among partners rather than which traits the participants possessed themselves, so each confederate assistant also completed this survey. Item-level Pearson correlations were then conducted between participant and their interaction partner to yield a measure of personality similarity.

2. **Modified Avocation Activities Questionnaire**

(McManus et al., 2011). This measure assesses the frequency of engaging in various leisure activities outside of work or school, with some language and items modified to include newer technologies and activities that current students are more likely to engage in (see items below). For this study, we were interested in similarity in interests among partners, so each confederate assistant also completed this survey. Item-level Pearson correlations were then conducted

between participant and their interaction partner to yield a measure of similarity in interests and activities.

Supplementary Table 1. Modified Avocation Activities Questionnaire

Please describe a little about your interests and activities outside your job.

How often do you:	every day	a few times a week	once a week	a few times a month	once a month or less	a few times a year	never
Listen to popular music							
Listen to classical music							
Go to popular music concerts							
Go to classical music concerts / opera							
Play a musical instrument							
Go to museums or art galleries							
Draw, paint, sculpt or do other arts or crafts							
Photography							
Read newspapers or magazines (paper or online)							
Read a novel							

Read non-fiction books (not for work or study)							
Read poetry							
Write poetry, fiction or other literature (not for work)							
Go to the movies							
Go to the theatre (plays/musicals, etc)							
Acting or otherwise taking part in theatre							
Watching classical or modern ballet/dance							
Dance (any form)							
Play a sport							
Watch sports							
Hike, run, or walk outdoors (for pleasure or exercise)							
Go to the gym / work out at home							
Cook / bake							

Shop (for pleasure)							
Spend time on hobbies (excluding above activities)							
	-------------------- Most days for --------------------						
	4+ hours	2-4 hours	1-2 hours	1 hour or less	2-3 times a week	once a week	less often
Watch television							
Watch movies / videos / etc.							
Listen to radio (or stream music on spotify, etc.)							
Listen to podcasts							
Browse the internet (not for work)							
Play video games							

fMRI Measures

Naturalistic videos were selected using the following criteria: 1) they should cover a variety of topics and interests; 2) they should elicit different types of emotions in different people (e.g., one person may find a clip "sweet" while another finds it "sappy"); 3) they should be novel to a majority of participants; 4) the timing should be around 2-5 minutes per clip. Videos were collected from YouTube (www.youtube.com) and trimmed for timing and content in Adobe Premiere Pro (www.adobe.com/products/).

Supplementary Table 2. Naturalistic Videos

Video	Time	Description
Delano Grape Workers Strike	2:11	Documentary clip about Filipino and Mexican farm laborers on strike for better working conditions
Wedding Music Video	3:40	Country music love song over clips of various weddings and receptions
"Nathan For You" Comedy Fake Dating Show	3:28	Satirical comedy clip where a man sets up a fake dating reality show
"Triumph over Adversity" Ballerina Aesha Ash	3:16	Documentary clip about a Black ballerina promoting positive images of women of color in her community
Penguin Nature Documentary	1:50	Documentary clip about emperor penguin chicks fighting off a petrol attack
Shark Tank Australia Reality Show	4:17	Reality show clip about investors giving money to two women to begin a special needs child care business
College Basketball Game	3:08	March Madness basketball tournament game clip where a team makes a surprise comeback
"The Onion" News on Nigeria's new President	2:41	Satirical news panel clip where journalists do not know anything about the topic they are meant to be experts in
Cooking Show	3:32	Cooking show clip where Rachael Ray shows how to make buffalo chicken bites
Mars Rover Simulator	5:19	Video showing a simulation of how a rover will land on Mars and collect data and images
America's Funniest Videos	1:12	Home videos of children getting scared by turkeys and people stepping on rakes
Fawlty Towers Sitcom	2:44	British sitcom clip where an old woman can't hear because she has turned her hearing aid off

fMRI Preprocessing Details

Results included in this manuscript come from preprocessing performed using fMRIPrep 20.2.5 (Esteban, Markiewicz, et al. (2018); Esteban, Blair, et al. (2018); RRID:SCR_016216), which is based on Nipype 1.6.1 (Gorgolewski et al. (2011); Gorgolewski et al. (2018); RRID:SCR_002502). The boilerplate text was automatically generated by fMRIPrep with the express intention that users should copy and paste this text into their manuscripts unchanged. It is released under the CC0 license.

Anatomical data preprocessing. A total of 1 T1-weighted (T1w) images were found within the input BIDS dataset.The T1-weighted (T1w) image was corrected for intensity non-uniformity (INU) with N4BiasFieldCorrection (Tustison et al. 2010), distributed with ANTs 2.3.3

(Avants et al. 2008, RRID:SCR_004757), and used as T1w-reference throughout the workflow. The T1w-reference was then skull-stripped with a Nipype implementation of the antsBrainExtraction.sh workflow (from ANTs), using MNI152NLin2009cAsym as target template. Brain tissue segmentation of cerebrospinal fluid (CSF), white-matter (WM) and gray-matter (GM) was performed on the brain-extracted T1w using fast (FSL 5.0.9, RRID:SCR_002823, Zhang, Brady, and Smith 2001). Brain surfaces were reconstructed using recon-all (FreeSurfer 6.0.1, RRID:SCR_001847, Dale, Fischl, and Sereno 1999), and the brain mask estimated previously was refined with a custom variation of the method to reconcile ANTs-derived and FreeSurfer-derived segmentations of the cortical gray-matter of Mindboggle (RRID:SCR_002438, Klein et al. 2017). Volume-based spatial normalization to two standard spaces (MNI152NLin2009cAsym, MNI152NLin6Asym) was performed through nonlinear registration with antsRegistration (ANTs 2.3.3), using brain-extracted versions of both T1w reference and the T1w template. The following templates were selected for spatial normalization: ICBM 152 Nonlinear Asymmetrical template version 2009c [Fonov et al. (2009), RRID:SCR_008796; TemplateFlow ID: MNI152NLin2009cAsym], FSL's MNI ICBM 152 non-linear 6th Generation Asymmetric Average Brain Stereotaxic Registration Model [Evans et al. (2012), RRID:SCR_002823; TemplateFlow ID: MNI152NLin6Asym].

Functional data preprocessing. For each of the 6 BOLD runs found per subject (across all tasks and sessions), the following preprocessing was performed. First, a reference volume and its skull-stripped version were generated using a custom methodology of fMRIPrep. A B0-nonuniformity map (or fieldmap) was estimated based on two (or more) echo-planar imaging (EPI) references with opposing phase-encoding directions, with 3dQwarp Cox and Hyde (1997) (AFNI 20160207). Based on the estimated susceptibility distortion, a corrected EPI (echo-planar imaging) reference was calculated for a more accurate co-registration with the anatomical reference. The BOLD reference was then co-registered to the T1w reference using bbregister

(FreeSurfer) which implements boundary-based registration (Greve and Fischl 2009). Co-registration was configured with six degrees of freedom. Head-motion parameters with respect to the BOLD reference (transformation matrices, and six corresponding rotation and translation parameters) are estimated before any spatiotemporal filtering using mcflirt (FSL 5.0.9, Jenkinson et al. 2002). BOLD runs were slice-time corrected to 0.56s (0.5 of slice acquisition range 0s-1.12s) using 3dTshift from AFNI 20160207 (Cox and Hyde 1997, RRID:SCR_005927). The BOLD time-series were resampled onto the following surfaces (FreeSurfer reconstruction nomenclature): fsaverage. The BOLD time-series (including slice-timing correction when applied) were resampled onto their original, native space by applying a single, composite transform to correct for head-motion and susceptibility distortions. These resampled BOLD time-series will be referred to as preprocessed BOLD in original space, or just preprocessed BOLD. The BOLD time-series were resampled into standard space, generating a preprocessed BOLD run in MNI152NLin2009cAsym space. First, a reference volume and its skull-stripped version were generated using a custom methodology of fMRIPrep. Grayordinates files (Glasser et al. 2013) containing 91k samples were also generated using the highest-resolution fsaverage as intermediate standardized surface space. Automatic removal of motion artifacts using independent component analysis (ICA-AROMA, Pruim et al. 2015) was performed on the preprocessed BOLD on MNI space time-series after removal of non-steady state volumes and spatial smoothing with an isotropic, Gaussian kernel of 6mm FWHM (full-width half-maximum). Corresponding "non-aggressively" denoised runs were produced after such smoothing. Additionally, the "aggressive" noise-regressors were collected and placed in the corresponding confounds file. Several confounding time-series were calculated based on the preprocessed BOLD: framewise displacement (FD), DVARS and three region-wise global signals. FD was computed using two formulations following Power (absolute sum of relative motions, Power et al. (2014)) and Jenkinson (relative root mean square displacement between affines, Jenkinson et al. (2002)). FD and DVARS are calculated for each functional run, both using their implementations

in Nipype (following the definitions by Power et al. 2014). The three global signals are extracted within the CSF, the WM, and the whole-brain masks. Additionally, a set of physiological regressors were extracted to allow for component-based noise correction (CompCor, Behzadi et al. 2007). Principal components are estimated after high-pass filtering the preprocessed BOLD time-series (using a discrete cosine filter with 128s cut-off) for the two CompCor variants: temporal (tCompCor) and anatomical (aCompCor). tCompCor components are then calculated from the top 2% variable voxels within the brain mask. For aCompCor, three probabilistic masks (CSF, WM and combined CSF+WM) are generated in anatomical space. The implementation differs from that of Behzadi et al. in that instead of eroding the masks by 2 pixels on BOLD space, the aCompCor masks are subtracted a mask of pixels that likely contain a volume fraction of GM. This mask is obtained by dilating a GM mask extracted from the FreeSurfer's aseg segmentation, and it ensures components are not extracted from voxels containing a minimal fraction of GM. Finally, these masks are resampled into BOLD space and binarized by thresholding at 0.99 (as in the original implementation). Components are also calculated separately within the WM and CSF masks. For each CompCor decomposition, the k components with the largest singular values are retained, such that the retained components' time series are sufficient to explain 50 percent of variance across the nuisance mask (CSF, WM, combined, or temporal). The remaining components are dropped from consideration. The head-motion estimates calculated in the correction step were also placed within the corresponding confounds file. The confound time series derived from head motion estimates and global signals were expanded with the inclusion of temporal derivatives and quadratic terms for each (Satterthwaite et al. 2013). Frames that exceeded a threshold of 0.5 mm FD or 1.5 standardised DVARS were annotated as motion outliers. All resamplings can be performed with a single interpolation step by composing all the pertinent transformations (i.e. head-motion transform matrices, susceptibility distortion correction when available, and co-registrations to anatomical and output spaces). Gridded (volumetric) resamplings were performed using antsApplyTransforms (ANTs), configured with Lanczos

interpolation to minimize the smoothing effects of other kernels (Lanczos 1964). Non-gridded (surface) resamplings were performed using mri_vol2surf (FreeSurfer). First, a reference volume and its skull-stripped version were generated using a custom methodology of fMRIPrep. A B0-nonuniformity map (or fieldmap) was estimated based on two (or more) echo-planar imaging (EPI) references with opposing phase-encoding directions, with 3dQwarp Cox and Hyde (1997) (AFNI 20160207). Based on the estimated susceptibility distortion, a corrected EPI (echo-planar imaging) reference was calculated for a more accurate co-registration with the anatomical reference. The BOLD reference was then co-registered to the T1w reference using bbregister (FreeSurfer) which implements boundary-based registration (Greve and Fischl 2009). Co-registration was configured with six degrees of freedom. Head-motion parameters with respect to the BOLD reference (transformation matrices, and six corresponding rotation and translation parameters) are estimated before any spatiotemporal filtering using mcflirt (FSL 5.0.9, Jenkinson et al. 2002). BOLD runs were slice-time corrected to 0.559s (0.5 of slice acquisition range 0s-1.12s) using 3dTshift from AFNI 20160207 (Cox and Hyde 1997, RRID:SCR_005927). The BOLD time-series were resampled onto the following surfaces (FreeSurfer reconstruction nomenclature): fsaverage. The BOLD time-series (including slice-timing correction when applied) were resampled onto their original, native space by applying a single, composite transform to correct for head-motion and susceptibility distortions. These resampled BOLD time-series will be referred to as preprocessed BOLD in original space, or just preprocessed BOLD. The BOLD time-series were resampled into standard space, generating a preprocessed BOLD run in MNI152NLin2009cAsym space. First, a reference volume and its skull-stripped version were generated using a custom methodology of fMRIPrep. Grayordinates files (Glasser et al. 2013) containing 91k samples were also generated using the highest-resolution fsaverage as intermediate standardized surface space. Automatic removal of motion artifacts using independent component analysis (ICA-AROMA, Pruim et al. 2015) was performed on the preprocessed BOLD on MNI space time-series after removal of non-steady state volumes and

spatial smoothing with an isotropic, Gaussian kernel of 6mm FWHM (full-width half-maximum). Corresponding "non-aggressively" denoised runs were produced after such smoothing. Additionally, the "aggressive" noise-regressors were collected and placed in the corresponding confounds file. Several confounding time-series were calculated based on the preprocessed BOLD: framewise displacement (FD), DVARS and three region-wise global signals. FD was computed using two formulations following Power (absolute sum of relative motions, Power et al. (2014)) and Jenkinson (relative root mean square displacement between affines, Jenkinson et al. (2002)). FD and DVARS are calculated for each functional run, both using their implementations in Nipype (following the definitions by Power et al. 2014). The three global signals are extracted within the CSF, the WM, and the whole-brain masks. Additionally, a set of physiological regressors were extracted to allow for component-based noise correction (CompCor, Behzadi et al. 2007). Principal components are estimated after high-pass filtering the preprocessed BOLD time-series (using a discrete cosine filter with 128s cut-off) for the two CompCor variants: temporal (tCompCor) and anatomical (aCompCor). tCompCor components are then calculated from the top 2% variable voxels within the brain mask. For aCompCor, three probabilistic masks (CSF, WM and combined CSF+WM) are generated in anatomical space. The implementation differs from that of Behzadi et al. in that instead of eroding the masks by 2 pixels on BOLD space, the aCompCor masks are subtracted a mask of pixels that likely contain a volume fraction of GM. This mask is obtained by dilating a GM mask extracted from the FreeSurfer's aseg segmentation, and it ensures components are not extracted from voxels containing a minimal fraction of GM. Finally, these masks are resampled into BOLD space and binarized by thresholding at 0.99 (as in the original implementation). Components are also calculated separately within the WM and CSF masks. For each CompCor decomposition, the k components with the largest singular values are retained, such that the retained components' time series are sufficient to explain 50 percent of variance across the nuisance mask (CSF, WM, combined, or temporal). The remaining components are dropped from consideration. The head-motion estimates calculated in the

correction step were also placed within the corresponding confounds file. The confound time series derived from head motion estimates and global signals were expanded with the inclusion of temporal derivatives and quadratic terms for each (Satterthwaite et al. 2013). Frames that exceeded a threshold of 0.5 mm FD or 1.5 standardised DVARS were annotated as motion outliers. All resamplings can be performed with a single interpolation step by composing all the pertinent transformations (i.e. head-motion transform matrices, susceptibility distortion correction when available, and co-registrations to anatomical and output spaces). Gridded (volumetric) resamplings were performed using antsApplyTransforms (ANTs), configured with Lanczos interpolation to minimize the smoothing effects of other kernels (Lanczos 1964). Non-gridded (surface) resamplings were performed using mri_vol2surf (FreeSurfer).

Many internal operations of fMRIPrep use Nilearn 0.6.2 (Abraham et al. 2014, RRID:SCR_001362), mostly within the functional processing workflow. For more details of the pipeline, see the section corresponding to workflows in fMRIPrep's documentation.

fMRI Data Quality Motion Check

Average motion was low (0.1-035 mm) and did not show associations with neural similarity or either of the interaction success outcomes. Additionally, we correlated framewise displacement between participants and their partner across the same volumes that neural similarity was calculated. This motion similarity measure was not associated with neural similarity, but it was included in all group level model analyses which also included neural similarity as extra assurance that links between neural similarity and interaction outcomes were not influenced by motion similarity.

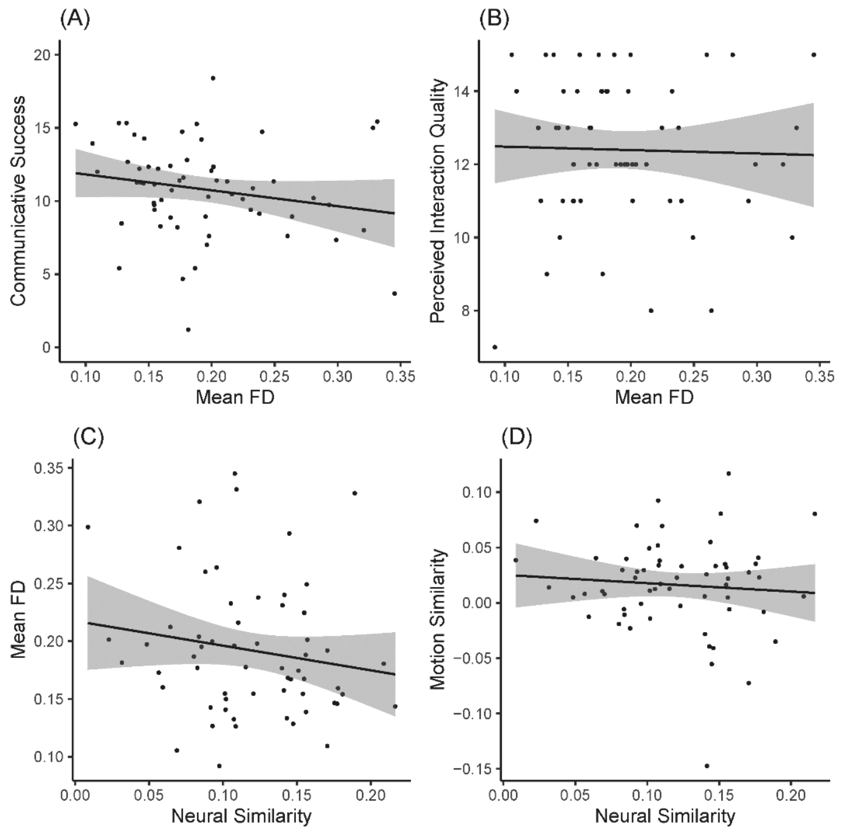

Supplementary Figure 1. MRI motion effects on neural similarity and interaction outcomes.

(A) Mean framewise displacement (FD) across the session is not associated with scores on the communicative success task (BF = 0.70, β = 0.64, p = 0.13). **(B)** Mean FD is not associated with self-reported perceived interaction quality (BF = 0.27, β = -0.05, p = 0.83). **(C)** Mean FD is not associated with neural similarity to partner (BF = 0.51, β = -0.01, p = 0.21). **(D)** Motion (FD) similarity to partner is not associated with neural similarity to partner (BF = 0.31, β = -0.003, p = 0.53).

Weighted Neural Similarity. Relative size was calculated for each parcel in the Shen atlas (ROI Voxels / Total Voxels) * 100. ROI correlations between participant and confederate partner were multiplied by this weight prior to averaging across all parcels. Using this weighted neural similarity measure did not materially change the pattern of results (**Supplementary Tables 9 & 13; Supplementary Figures 9 & 13**).

Measurement Comparison

This study included several measures of both social cognitive ability and partner similarity. **Supplementary Figure 2** shows correlation matrices for these measures. IRI and MRMS scales were the only measures significantly associated with each other. This mixed association between different social cognitive measures in particular fits with existing literature showing little overlap between measures of theory of mind (Warnell & Redcay, 2019).

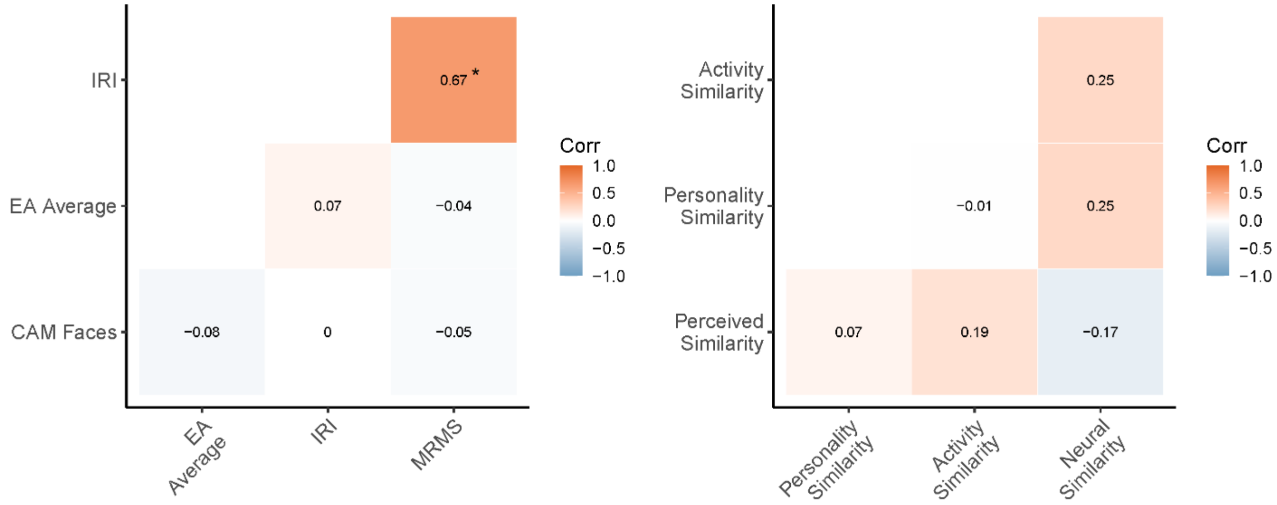

Supplementary Figure 2. Correlation matrices for social cognitive and similarity measures.

A. Social cognitive measures: interpersonal total scores (IRI), empathic accuracy performance averaged across three videos (EA Average), complex emotion recognition in faces task (CAM), and mind-reading motivation scale (MRMS). **B.** Similarity measures: activities and interests survey similarity, five-factor personality similarity, perceived similarity, and neural similarity. Asterisks denote r values with $p < 0.05$.

Supplementary Results

Supplementary Table 3. Social cognitive measures predicting communicative success.

Bayes Factors (BFs) and relative BFs compared to the confederate model alone.

Model	BF	BF_{model} / $BF_{confederate}$
Confederate	6.67	1
IRI + Confederate	5.30	0.79
MRMS + Confederate	3.84	0.58
Face Task + IRI + Confederate	2.14	0.32
Face Task + Confederate	2.10	0.31
IRI + MRMS + Confederate	2.08	0.31
EA + IRI + Confederate	1.96	0.29
EA + Confederate	1.83	0.27
Face Task + MRMS + Confederate	1.56	0.23
EA + MRMS + Confederate	1.45	0.22
Face Task + IRI + MRMS + Confederate	0.99	0.15
Face Task + EA + IRI + Confederate	0.94	0.14
EA + IRI + MRMS + Confederate	0.92	0.14
Face Task + EA + Confederate	0.82	0.12
Face Task + EA + MRMS + Confederate	0.70	0.10
Face Task + EA + IRI + MRMS + Confederate	0.49	0.07

Supplementary Figure 3. Social cognitive measures predicting communicative success.
Interpersonal reactivity scores (IRI) were the strongest predictor of communicative success, followed by mind-reading motivation scores (MRMS). All models included the effect of confederate. Note: the x-axis is not linear.

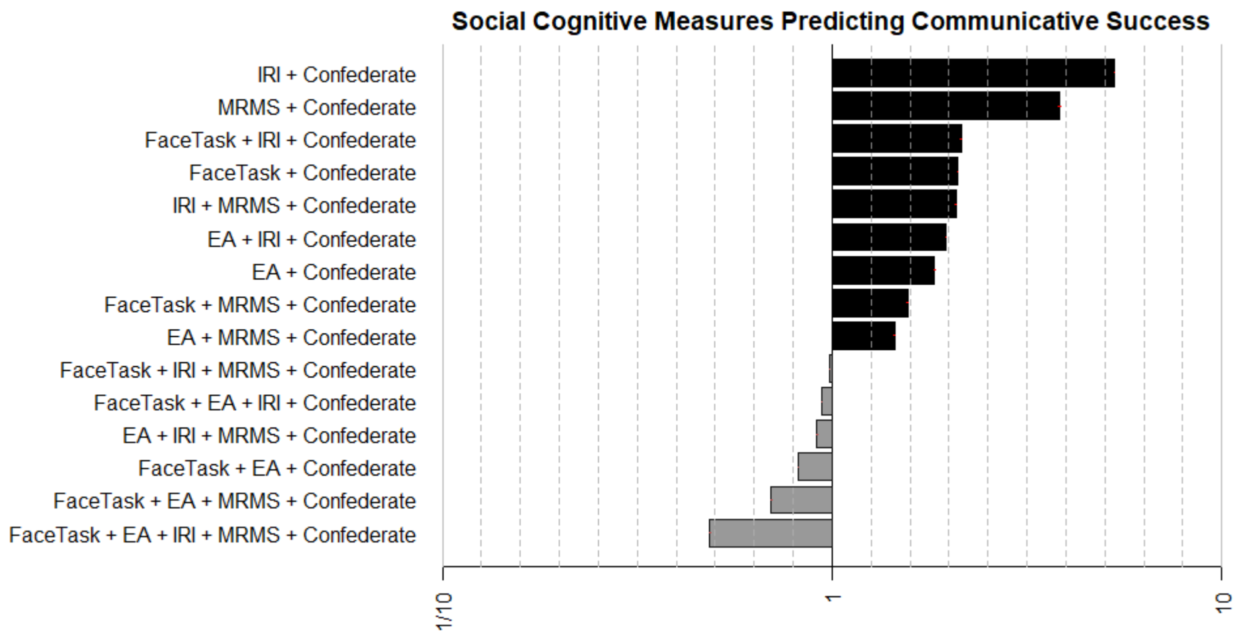

Supplementary Table 4. Similarity measures predicting communicative success.
Bayes Factors (BFs) and relative BFs compared to the covariate model alone.

Model	BF	BF_{model} / $BF_{covariates}$
Neural Similarity + Covariates	6.16	3.28
Activity Similarity + Neural Similarity + Covariates	3.63	1.93
Perceived Similarity + Neural Similarity + Covariates	3.02	1.61
Personality Similarity + Neural Similarity + Covariates	2.70	1.44
Covariates	1.88	1
Activity Similarity + Perceived Similarity + Neural Similarity + Covariates	1.78	0.95
Activity Similarity + Personality Similarity + Neural Similarity + Covariates	1.73	0.92
Personality Similarity + Perceived Similarity + Neural Similarity + Covariates	1.45	0.77
Activity Similarity + Covariates	1.30	0.69

Activity Similarity + Personality Similarity + Perceived Similarity + Neural Similarity + Covariates	0.91	0.48
Personality Similarity + Covariates	0.89	0.47
Perceived Similarity + Covariates	0.85	0.45
Activity Similarity + Personality Similarity + Covariates	0.69	0.37
Activity Similarity + Perceived Similarity + Covariates	0.63	0.34
Personality Similarity + Perceived Similarity + Covariates	0.45	0.24
Activity Similarity + Personality Similarity + Perceived Similarity + Covariates	0.36	0.19

Supplementary Figure 4. Similarity measures predicting communicative success. Neural similarity was the strongest predictor of communicative success. All models included the effect of confederate as well as the number of runs of MRI data and the effect of motion similarity. Note: the x-axis is not linear.

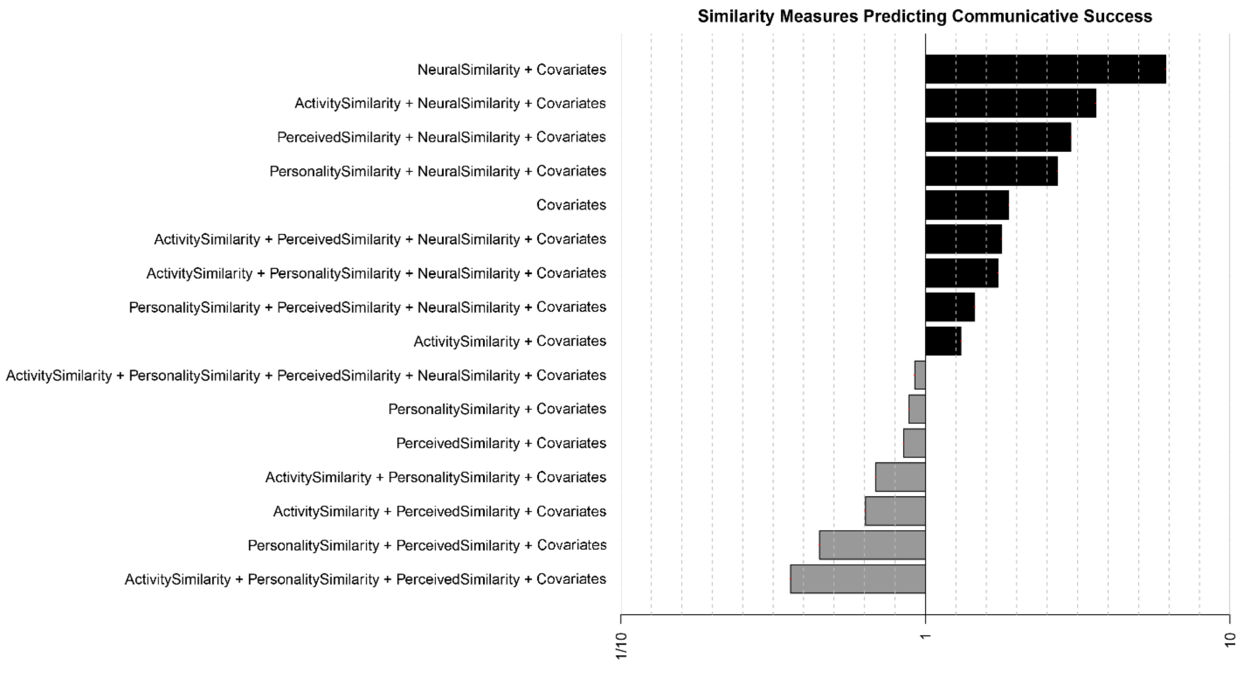

Supplementary Table 5. Social cognitive measures predicting communicative success (edited Telewave scores).
Bayes Factors (BFs) and relative BFs compared to the confederate model alone.

Model	BF	BF_{model} / $BF_{confederate}$
Confederate	7.73	1.00
IRI + Confederate	7.09	0.92
MRMS + Confederate	5.03	0.65
Face Task + IRI + Confederate	2.85	0.37
IRI + MRMS + Confederate	2.78	0.36
EA + IRI + Confederate	2.61	0.34
Face Task + Confederate	2.45	0.32
EA + Confederate	2.11	0.27
Face Task + MRMS + Confederate	2.05	0.26
EA + MRMS + Confederate	1.86	0.24
Face Task + IRI + MRMS + Confederate	1.32	0.17
Face Task + EA + IRI + Confederate	1.26	0.16
EA + IRI + MRMS + Confederate	1.23	0.16
Face Task + EA + Confederate	0.96	0.12
Face Task + EA + MRMS + Confederate	0.90	0.12
Face Task + EA + IRI + MRMS + Confederate	0.65	0.08

Supplementary Figure 5. Social cognitive measures predicting communicative success (edited Telewave scores).
Interpersonal reactivity scores (IRI) were the strongest predictor of communicative success using the edited scores with clue changes removed, followed by mind-reading motivation scores (MRMS). All models included the effect of confederate. Note: the x-axis is not linear.

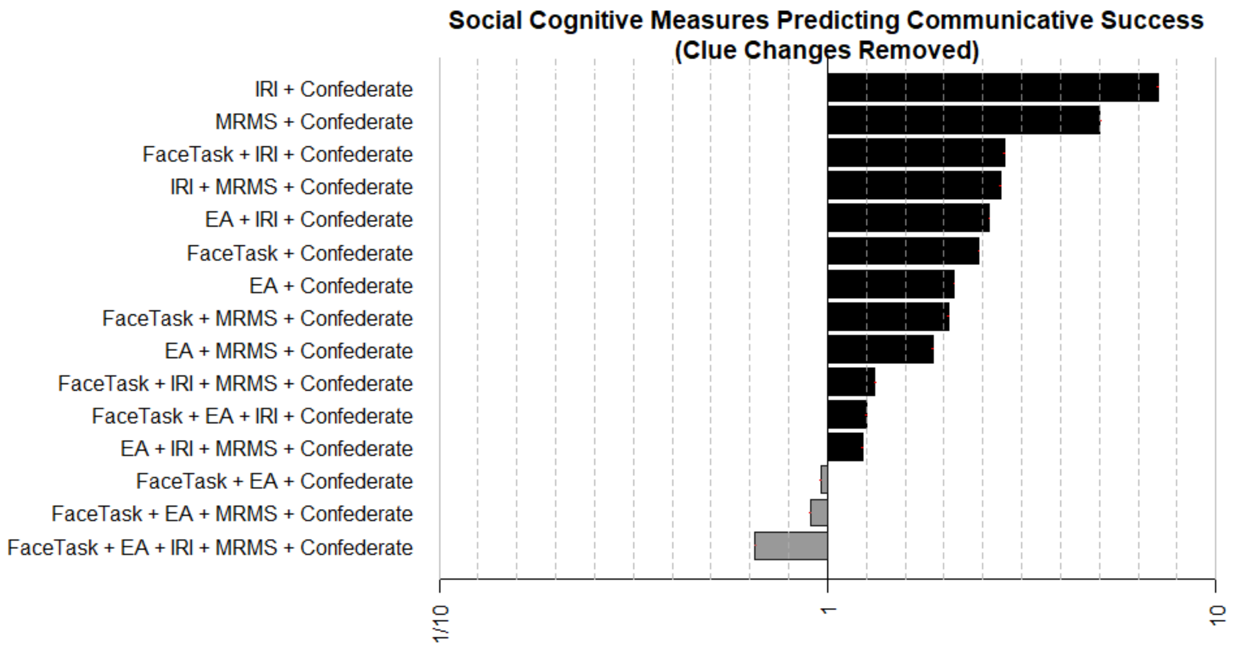

Supplementary Table 6. Similarity measures predicting communicative success (edited Telewave scores).

Bayes Factors (BFs) and relative BFs compared to the covariate model alone. All models included the effect of confederate as well as the number of runs of MRI data and the effect of motion similarity.

Model	BF	BF_{model} / $BF_{covariates}$
Neural Similarity + Covariates	12.44	3.94
Activity Similarity + Neural Similarity + Covariates	7.61	2.41
Perceived Similarity + Neural Similarity + Covariates	5.42	1.72
Personality Similarity + Neural Similarity + Covariates	5.38	1.70
Activity Similarity + Personality Similarity + Neural Similarity + Covariates	3.58	1.14
Activity Similarity + Perceived Similarity + Neural Similarity + Covariates	3.44	1.09
Covariates	3.15	1.00

Personality Similarity + Perceived Similarity + Neural Similarity + Covariates	2.57	0.82
Activity Similarity + Covariates	2.31	0.73
Activity Similarity + Personality Similarity + Perceived Similarity + Neural Similarity + Covariates	1.75	0.55
Personality Similarity + Covariates	1.50	0.48
Perceived Similarity + Covariates	1.38	0.44
Activity Similarity + Personality Similarity + Covariates	1.24	0.39
Activity Similarity + Perceived Similarity + Covariates	1.14	0.36
Personality Similarity + Perceived Similarity + Covariates	0.74	0.24
Activity Similarity + Personality Similarity + Perceived Similarity + Covariates	0.67	0.21

Supplementary Figure 6. Similarity measures predicting communicative success (edited Telewave scores).
Neural similarity was the strongest predictor of communicative success. All models included the effect of confederate as well as the number of runs of MRI data and the effect of motion similarity. Note: the x-axis is not linear.

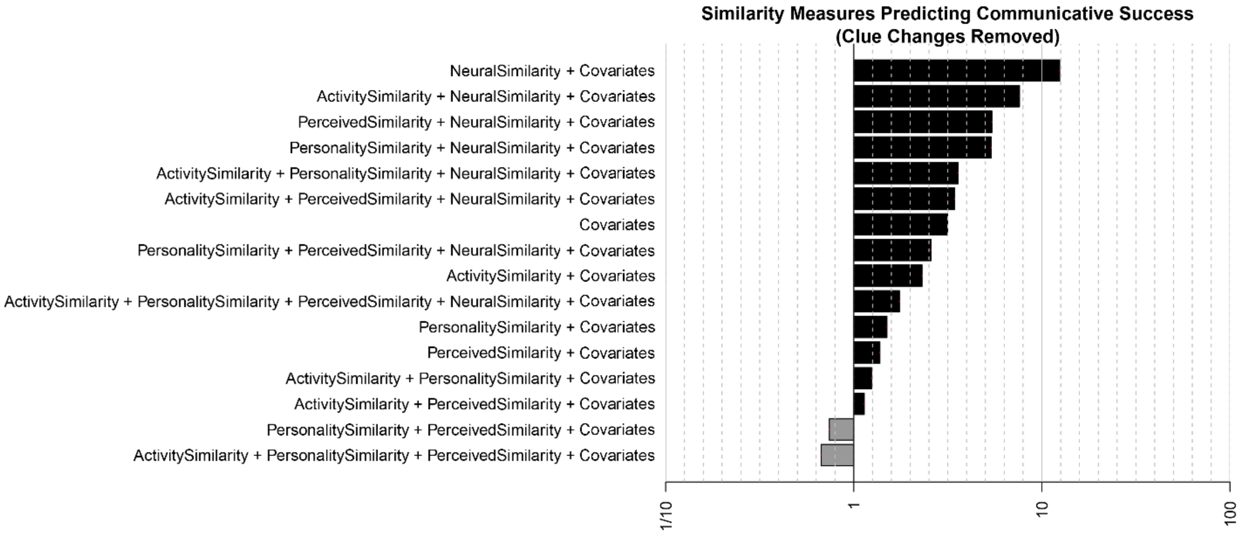

Supplementary Table 7. Main effects and interaction models predicting communicative success (edited Telewave scores).

Bayes Factors (BFs) and relative BFs compared to the covariate model alone. All models included the effect of confederate as well as the number of runs of MRI data and the effect of motion similarity.

Model	BF	BF_{model} / $BF_{covariates}$
IRI + Neural Similarity + Covariates	39.06	12.42
IRI + Neural Similarity + IRI:Neural Similarity + Covariates	29.64	9.43
Neural Similarity + Covariates	12.47	3.97
IRI + Covariates	3.76	1.20
Covariates	3.14	1.00

Supplementary Figure 7. Main effects and interaction models predicting communicative success (edited Telewave scores).

The joint main effects model was again the strongest predictor of communicative success using the edited scores with clue changes removed, followed by the interaction model, although these models were very close to equal evidence to explain the outcome. All models included the effect of confederate as well as the number of runs of MRI data and the effect of motion similarity. Note: the x-axis is not linear.

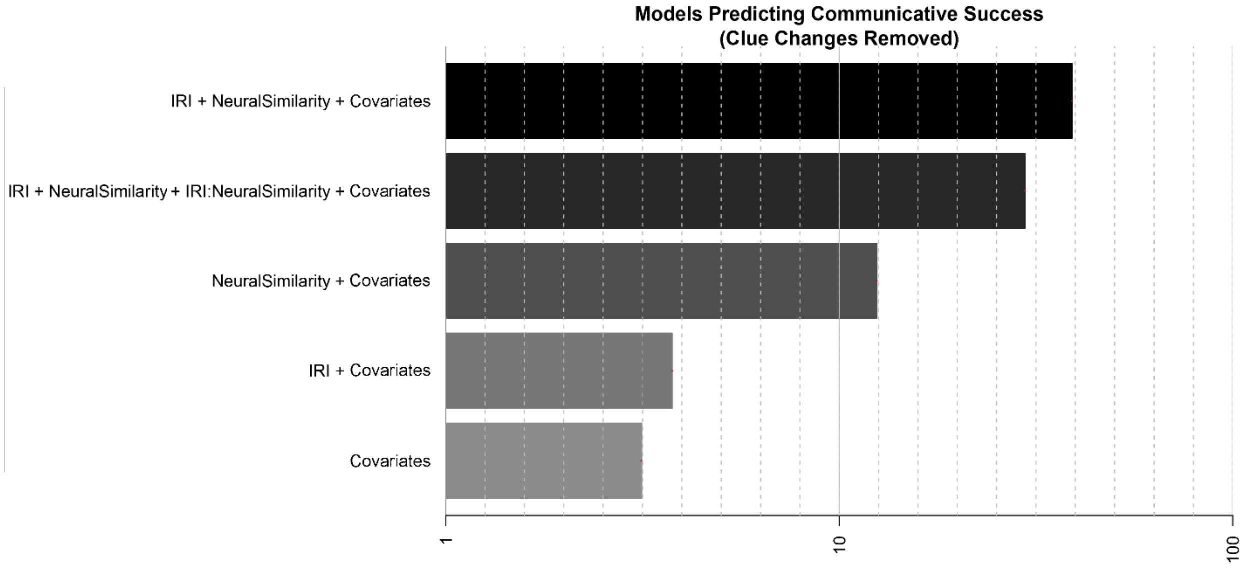

Supplementary Table 8. Social cognitive measures predicting communicative success (edited Face Task).

Bayes Factors (BFs) and relative BFs compared to the confederate model alone.

Model	BF	BF_{model} / $BF_{confederate}$

Confederate	7.01	1.00
IRI + Confederate	5.93	0.85
MRMS + Confederate	3.79	0.54
IRI + MRMS + Confederate	2.07	0.29
EA + Confederate	1.96	0.28
EA + IRI + Confederate	1.94	0.28
EA + MRMS + Confederate	1.43	0.20
Face Task + Confederate	0.97	0.14
EA + IRI + MRMS + Confederate	0.91	0.13
Face Task + IRI + Confederate	0.76	0.11
Face Task + MRMS + Confederate	0.59	0.08
Face Task + IRI + MRMS + Confederate	0.32	0.04
Face Task + EA + IRI + Confederate	0.30	0.04
Face Task + EA + Confederate	0.28	0.04
Face Task + EA + MRMS + Confederate	0.23	0.03
Face Task + EA + IRI + MRMS + Confederate	0.15	0.02

Supplementary Figure 8. Social cognitive measures predicting communicative success (edited Face Task).
Interpersonal reactivity scores (IRI) remained the strongest predictor of communicative success with the subject who only completed 50% of the face task trials removed. All models included the effect of confederate. Note: the x-axis is not linear.

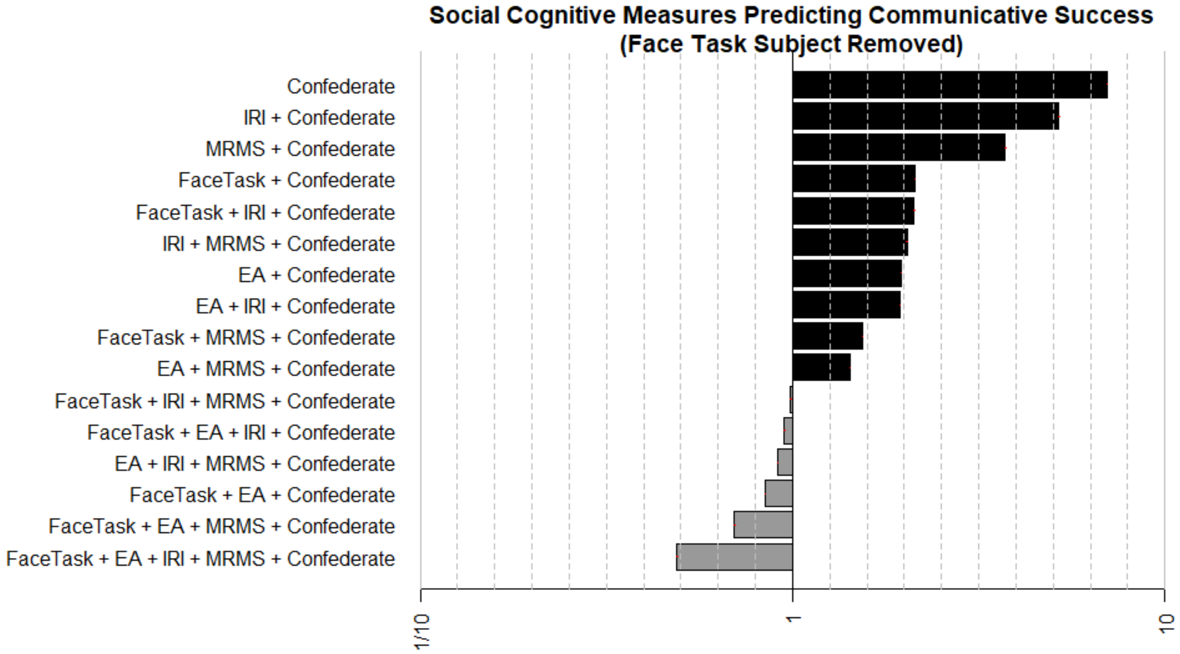

Supplementary Table 9. Similarity measures predicting communicative success (Weighted Neural Similarity).

Bayes Factors (BFs) and relative BFs compared to the covariate model alone. All models included the effect of confederate as well as the number of runs of MRI data and the effect of motion similarity.

Model	BF	BF_{model} / $BF_{covariates}$
Neural Similarity (Weighted) + Covariates	7.33	3.91
Activity Similarity + Neural Similarity (Weighted) + Covariates	4.28	2.29
Perceived Similarity + Neural Similarity (Weighted) + Covariates	3.73	1.99
Personality Similarity + Neural Similarity (Weighted) + Covariates	3.19	1.70
Activity Similarity + Perceived Similarity + Neural Similarity (Weighted) + Covariates	2.16	1.15
Activity Similarity + Personality Similarity + Neural Similarity (Weighted) + Covariates	2.04	1.09
Covariates	1.87	1
Personality Similarity + Perceived Similarity + Neural Similarity (Weighted) + Covariates	1.77	0.95

Activity Similarity + Covariates	1.30	0.69
Activity Similarity + Personality Similarity + Perceived Similarity + Neural Similarity (Weighted) + Covariates	1.10	0.59
Personality Similarity + Covariates	0.89	0.47
Perceived Similarity + Covariates	0.85	0.45
Activity Similarity + Personality Similarity + Covariates	0.69	0.37
Activity Similarity + Perceived Similarity + Covariates	0.64	0.34
Personality Similarity + Perceived Similarity + Covariates	0.45	0.24
Activity Similarity + Personality Similarity + Perceived Similarity + Covariates	0.36	0.19

Supplementary Figure 9. Similarity measures predicting communicative success (Weighted Neural Similarity).
Weighted neural similarity was the strongest predictor of communicative success. All models included the effect of confederate as well as the number of runs of MRI data and the effect of motion similarity. Note: the x-axis is not linear.

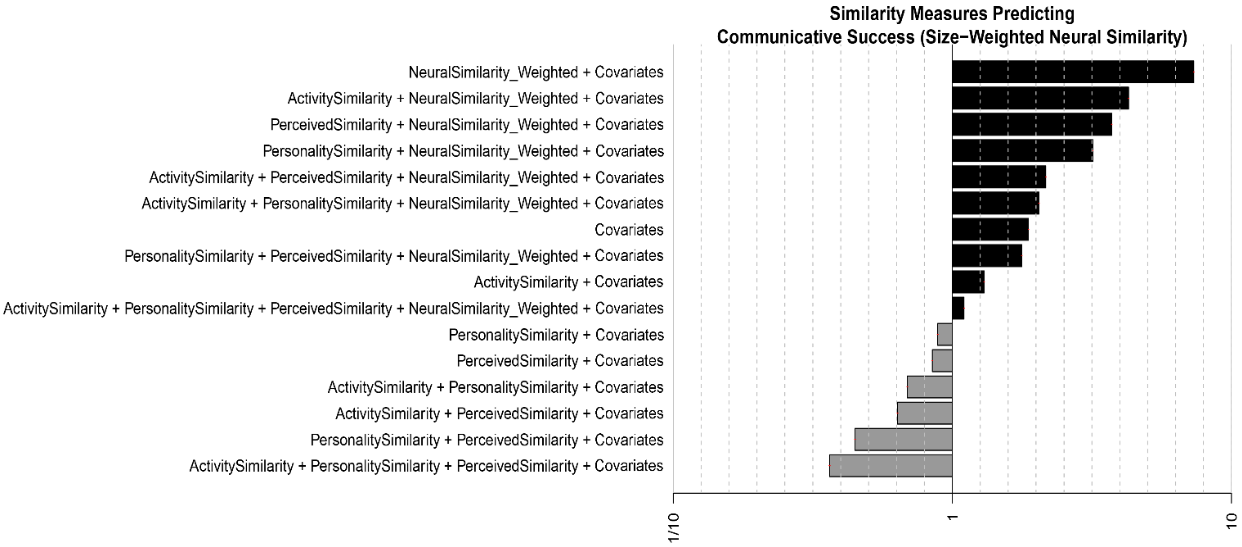

Supplementary Table 10. Cognitive and Emotional Empathy (IRI) Subscales predicting communicative success.

Model	BF	BF_{model} / $BF_{confederate}$
Confederate	6.67	1
Emotional Empathy (IRI) Subscale + Confederate	6.01	0.90
Cognitive Empathy (IRI) Subscale + Confederate	2.86	0.43
Emotional Empathy + Cognitive Empathy + Confederate	2.14	0.32

Supplementary Figure 10. Cognitive and Emotional Empathy (IRI) Subscales predicting communicative success.
Note: the x-axis is not linear.

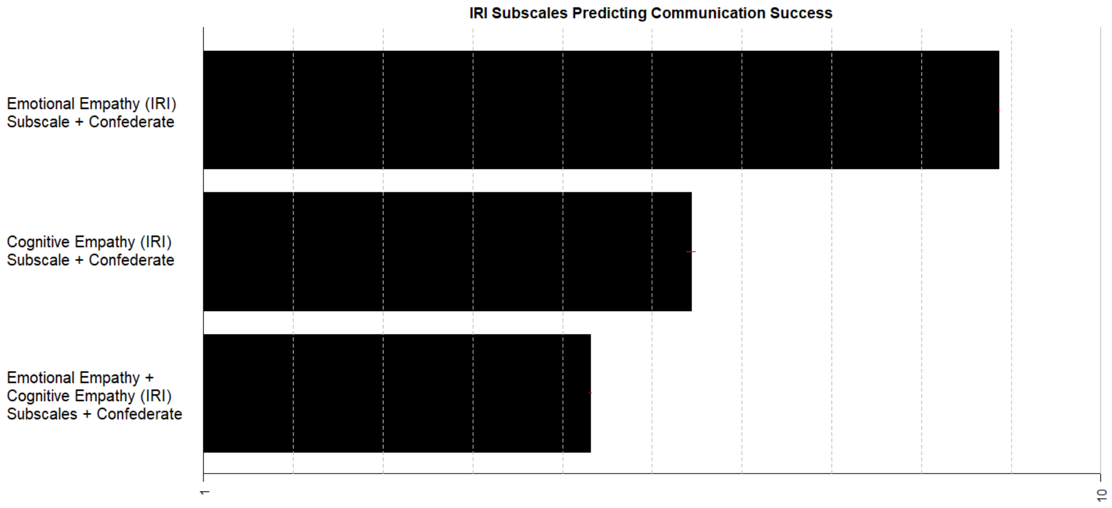

Supplementary Table 11. Main effects and interaction model predicting communicative success (Emotional Empathy IRI Subscale).

Model	BF	BF_{model} / $BF_{covariates}$
Emotional Empathy (IRI Subscale) + Neural Similarity + Covariates	19.12	10.22
Emotional Empathy (IRI Subscale) + Neural Similarity + Emotional Empathy:Neural Similarity + Covariates	10.22	5.46
Neural Similarity + Covariates	6.18	3.30
Emotional Empathy + Covariates	2.07	1.11
Covariates	1.87	1

Supplementary Figure 11. Main effects and interaction model predicting communicative success (Emotional Empathy IRI Subscale).

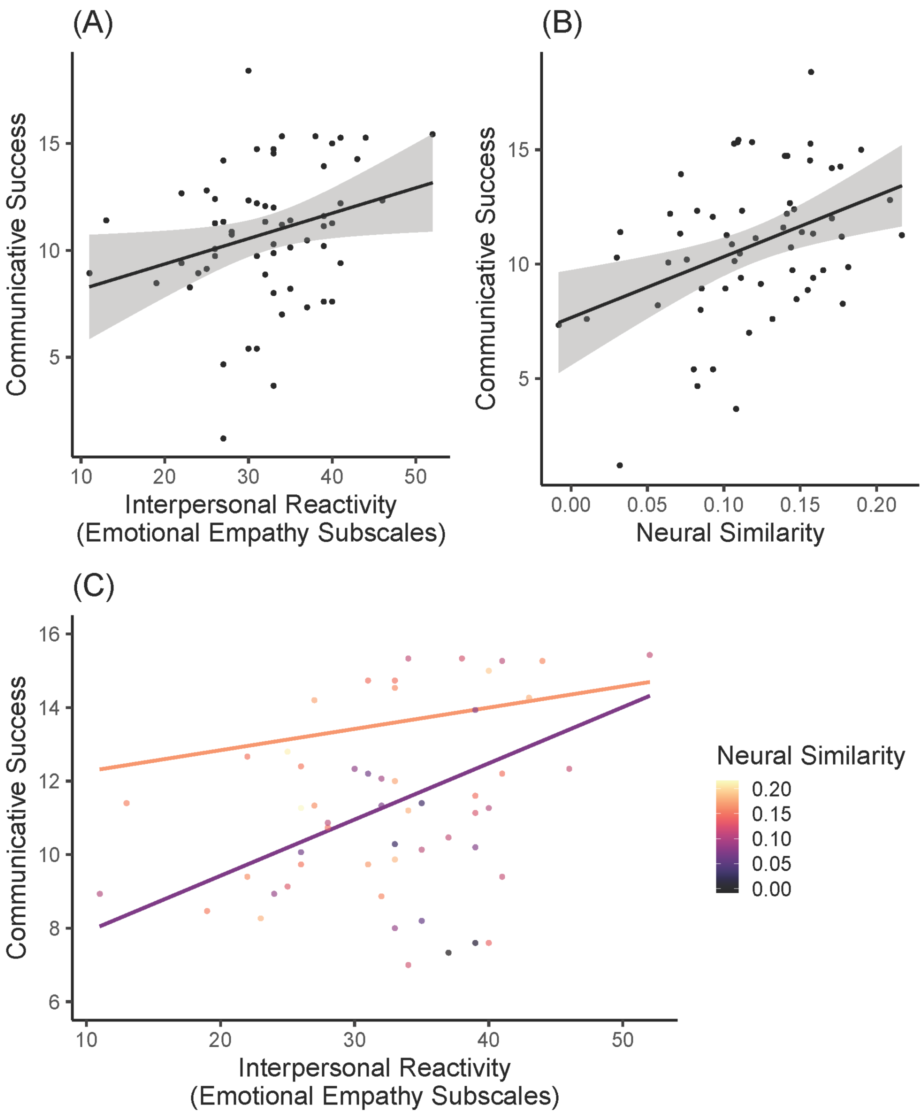

Supplementary Table 12. Social cognitive measures predicting perceived interaction quality.
Bayes Factors (BFs) and relative BFs compared to the confederate model alone.

Model	BF	BF_{model} / $BF_{confederate}$
MRMS + Confederate	2.15	11.09
EA + MRMS + Confederate	0.73	3.79

IRI + MRMS + Confederate	0.71	3.68
Face Task + MRMS + Confederate	0.70	3.61
IRI + Confederate	0.36	1.86
EA + IRI + MRMS + Confederate	0.31	1.57
Face Task + EA + MRMS + Confederate	0.30	1.52
Face Task + IRI + MRMS + Confederate	0.28	1.47
Confederate	0.19	1
EA + IRI + Confederate	0.16	0.84
Face Task + EA + IRI + MRMS + Confederate	0.14	0.72
Face Task + IRI + Confederate	0.14	0.70
Face Task + EA + IRI + Confederate	0.07	0.38
Face Task + Confederate	0.06	0.31
EA + Confederate	0.06	0.30
Face Task + EA + Confederate	0.03	0.14

Supplementary Figure 12. Social cognitive measures predicting perceived interaction quality.
Mind-reading motivation scores (MRMS) were the strongest predictor of perceived interaction quality, and the only measure with a BF > 1. All models included the effect of confederate. Note: the x-axis is not linear.

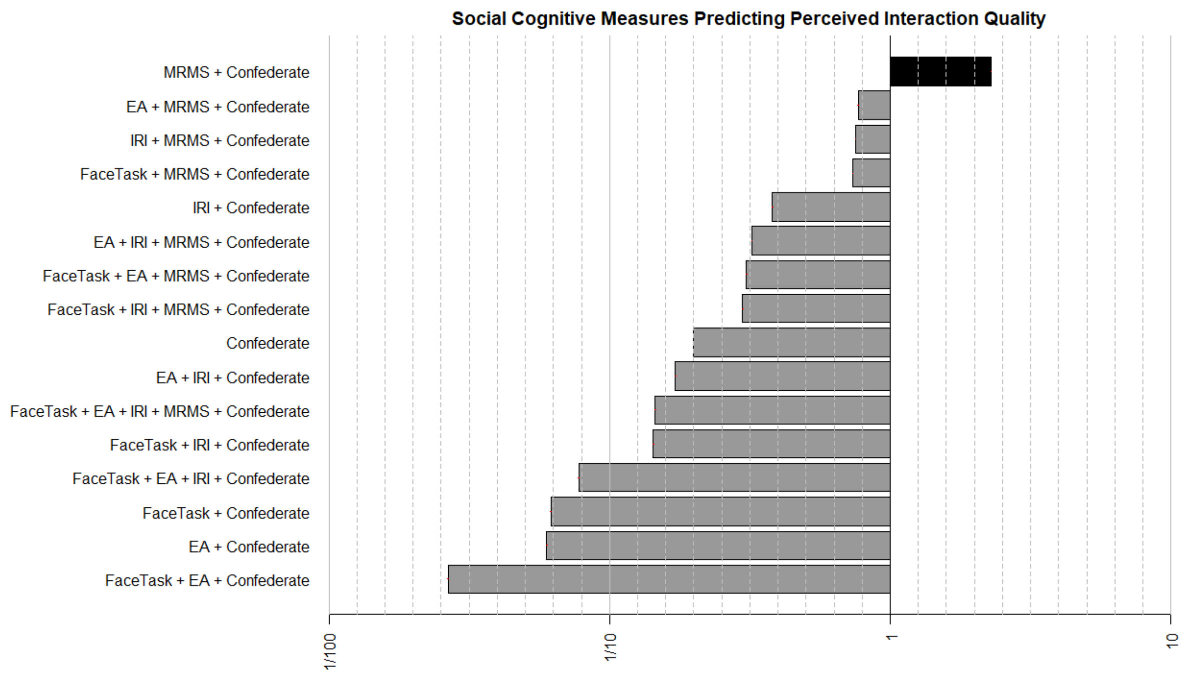

Supplementary Table 13. Similarity measures predicting perceived interaction quality.

Bayes Factors (BFs) and relative BFs compared to the covariate model alone.

Model	BF	BF_{model} / $BF_{covariates}$
Perceived Similarity + Covariates	257.99	9849.29
Activity Similarity + Perceived Similarity + Covariates	92.7	3538.84
Perceived Similarity + Neural Similarity + Covariates	89.98	3435.18
Personality Similarity + Perceived Similarity + Covariates	81.43	3108.73
Activity Similarity + Perceived Similarity + Neural Similarity + Covariates	35.79	1366.3
Activity Similarity + Personality Similarity + Perceived Similarity + Covariates	32.84	1253.66
Personality Similarity + Perceived Similarity + Neural Similarity + Covariates	31.74	1211.79
Activity Similarity + Personality Similarity + Perceived Similarity + Neural Similarity + Covariates	13.87	529.61

Covariates	0.03	1
Activity Similarity + Covariates	0.02	0.85
Activity Similarity + Neural Similarity + Covariates	0.02	0.72
Neural Similarity + Covariates	0.02	0.68
Personality Similarity + Covariates	0.01	0.49
Activity Similarity + Personality Similarity + Covariates	0.01	0.46
Activity Similarity + Personality Similarity + Neural Similarity + Covariates	0.01	0.43
Personality Similarity + Neural Similarity + Covariates	0.01	0.38

Supplementary Figure 13. Similarity measures predicting perceived interaction quality.

Perceived similarity was the strongest predictor of perceived interaction quality. All models included the effect of confederate as well as the number of runs of MRI data and the effect of motion similarity. Note: the x-axis is not linear.

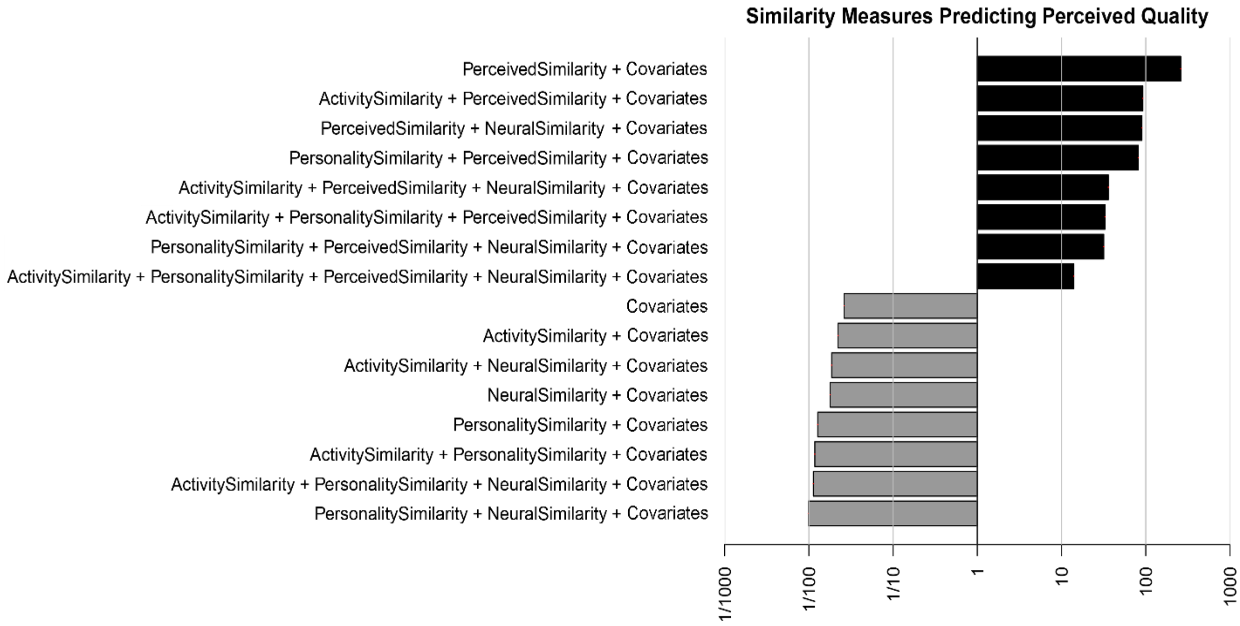

Supplementary Table 14. Social cognitive measures predicting perceived interaction quality (edited Face Task).

Bayes Factors (BFs) and relative BFs compared to the confederate model alone.

Model	BF	BF_{model} $/BF_{confederate}$
MRMS + Confederate	1.31	4.95
EA + MRMS + Confederate	0.46	1.73
IRI + MRMS + Confederate	0.45	1.72
IRI + Confederate	0.30	1.13
Confederate	0.26	1.00
Face Task + MRMS + Confederate	0.21	0.79
EA + IRI + MRMS + Confederate	0.20	0.74
EA + IRI + Confederate	0.13	0.49
EA + Confederate	0.08	0.29
Face Task + EA + MRMS + Confederate	0.07	0.28
Face Task + IRI + MRMS + Confederate	0.07	0.28
Face Task + IRI + Confederate	0.05	0.20
Face Task + Confederate	0.04	0.14
Face Task + EA + IRI + MRMS + Confederate	0.03	0.13
Face Task + EA + IRI + Confederate	0.02	0.09
Face Task + EA + Confederate	0.01	0.04

Supplementary Figure 14. Social cognitive measures predicting perceived interaction quality (edited Face Task).
Mind-reading motivation scores (MRMS) were the strongest predictor of communicative success and the only measure with a BF > 1 after removing the subject who only completed 50% of the face task trials. All models included the effect of confederate. Note: the x-axis is not linear.

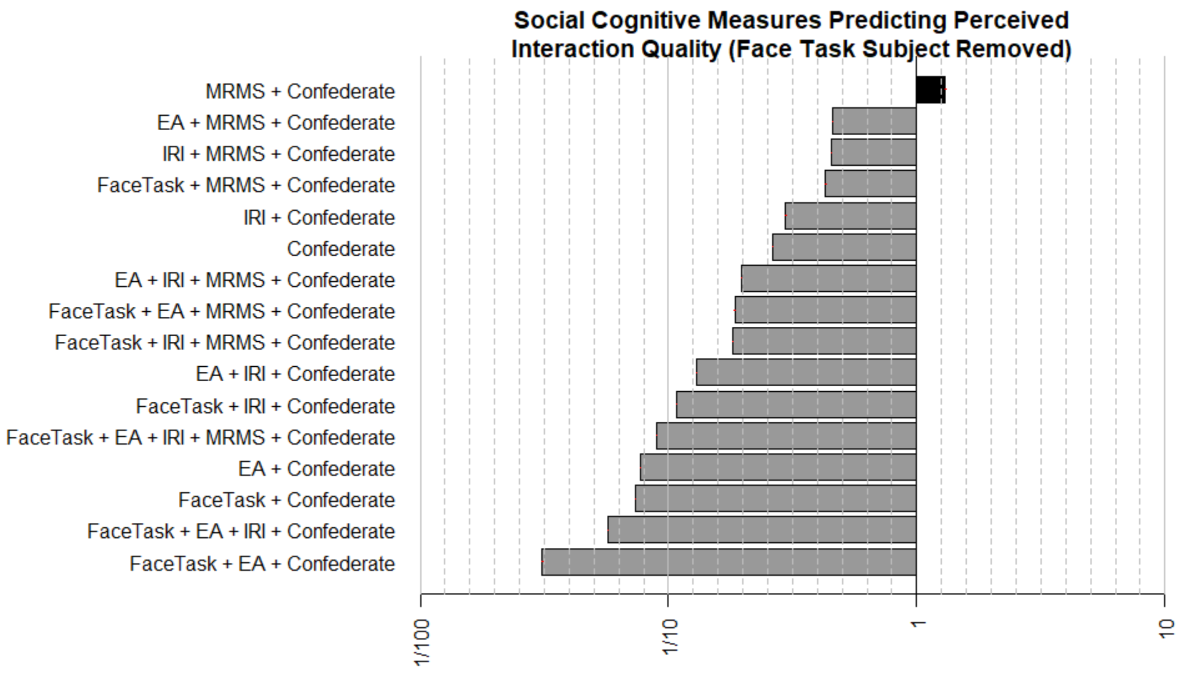

Supplementary Table 15. Similarity measures predicting perceived interaction quality (Weighted Neural Similarity).

Bayes Factors (BFs) and relative BFs compared to the covariate model alone. All models included the effect of confederate as well as the number of runs of MRI data and the effect of motion similarity.

Model	BF	BF_{model} / $BF_{covariates}$
Perceived Similarity + Covariates	259.49	9864.72
Activity Similarity + Perceived Similarity + Covariates	92.79	3527.47
Perceived Similarity + Neural Similarity (Weighted) + Covariates	90.68	3447.20
Personality Similarity + Perceived Similarity + Covariates	81.28	3089.98
Activity Similarity + Perceived Similarity + Neural Similarity (Weighted) + Covariates	36.01	1368.91
Activity Similarity + Personality Similarity + Perceived Similarity + Covariates	32.85	1248.61
Personality Similarity + Perceived Similarity + Neural Similarity (Weighted) + Covariates	31.80	1208.71

Activity Similarity + Personality Similarity + Perceived Similarity + Neural Similarity (Weighted) + Covariates	14.02	532.91
Covariates	0.03	1.00
Activity Similarity + Covariates	0.02	0.84
Activity Similarity + Neural Similarity (Weighted) + Covariates	0.02	0.81
Neural Similarity (Weighted) + Covariates	0.02	0.76
Activity Similarity + Personality Similarity + Neural Similarity (Weighted) + Covariates	0.01	0.49
Personality Similarity + Covariates	0.01	0.48
Activity Similarity + Personality Similarity + Covariates	0.01	0.45
Personality Similarity + Neural Similarity (Weighted) + Covariates	0.01	0.42

Supplementary Figure 15. Similarity measures predicting perceived interaction quality (Weighted Neural Similarity).
Perceived similarity was the strongest predictor of perceived interaction quality. All models included the effect of confederate as well as the number of runs of MRI data and the effect of motion similarity. Note: the x-axis is not linear.

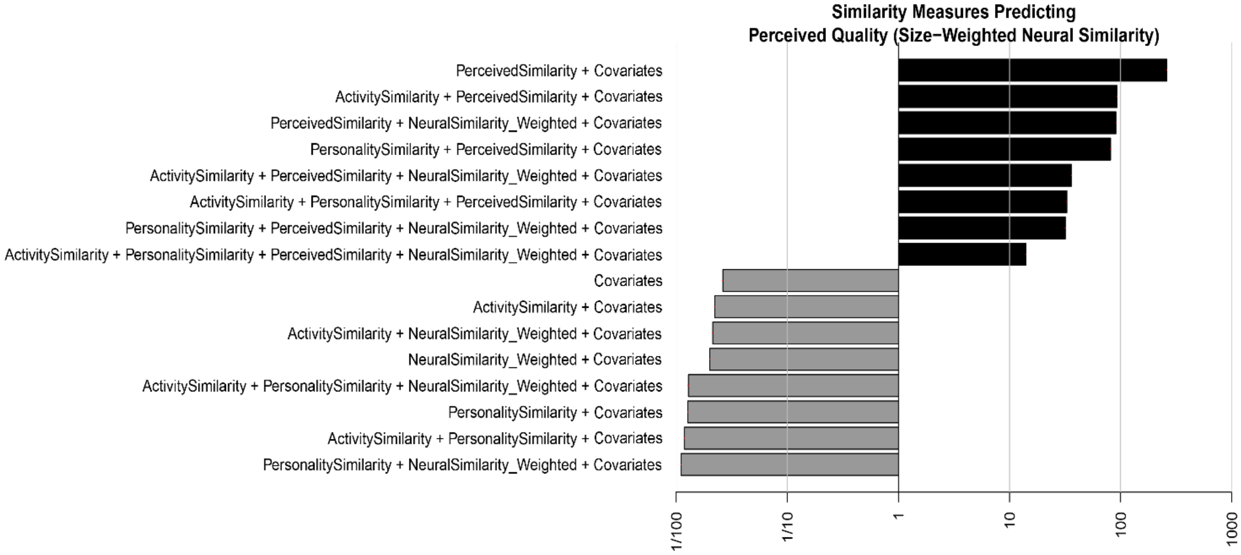

Supplementary Table 16. Brain Regions Predicting Communicative Success.
The following regions resulted in strong (BF > 10) or moderate evidence (BF > 3) against the null hypothesis when added to the main effects model in place of average neural similarity.

Parcel Number	Network	x	y	z	Region (Harvard-Oxford Cortical and Subcortical Structural Atlas)	BF	BF_{model} /$BF_{confederate}$
8	2	44.6	46.2	-4.9	Frontal Pole	143.34	76.65
21	2	55.4	9.6	22.2	Precentral Gyrus	74.23	39.7
194	1	-49.3	-4.7	-37.4	Inferior Temporal Gyrus: Anterior	41.25	22.06
153	1	-32	20.5	-16	Frontal Orbital Cortex	38.82	20.76
10	1	8.4	53.3	23.9	Superior Frontal Gyrus	23.43	12.53
71	8	41.6	-45.7	-22.6	Temporal Occipital Fusiform Cortex	19.04	10.18
16	1	53.6	24.8	0.9	Inferior Frontal Gyrus: para triangularis	14.77	7.9
195	5	-37.8	-13.2	-29.3	Temporal Pole	14.61	7.81
223	3	-5	-36	32	Cingulate Gyrus: Posterior Division	13.88	7.42
190	1	-57.6	-6.4	-22.7	Middle Temporal Gyrus: Anterior	13.66	7.31
219	1	-6	34.1	26.3	Paracingulate Gyrus	13	6.95
75	6	18.9	-81.8	41.5	Lateral Occipital Cortex: Superior	11.59	6.2
196	2	-51.8	-18.2	-28.8	Inferior Temporal Gyrus: Posterior	11.18	5.98
22	2	40	17.6	29.2	Middle Frontal Gyrus	11.09	5.93
251	4	-10.3	-37.7	-25.1	Cerebellum	10.66	5.7
192	1	-57.8	-47.5	5.2	Middle Temporal Gyrus: Temporo-occipital	10.65	5.69
85	3	5.1	-38.9	27	Cingulate Gyrus: Posterior	10.05	5.38
227	3	-7.5	-42.1	13.3	Cingulate Gyrus: Posterior	9.26	4.95
146	4	-27.3	34.1	36.4	Middle Frontal Gyrus	9.13	4.88
12	1	14.3	36.9	48.9	Frontal Pole	9.08	4.86
53	1	52.8	10.9	-21.8	Temporal Pole	9.03	4.83

63	5	61.8	-23.8	-2.8	Superior Temporal Gyrus: Posterior	8.64	4.62
65	1	59.3	-43.8	8.5	Middle Temporal Gyrus: Temporo-occipital	8.57	4.58
246	2	-42.6	-63.7	-46.3	Cerebellum	7.77	4.16
124	4	26.6	6.3	0.1	Putamen	7.63	4.08
126	4	10.5	-26.8	-2.2	Thalamus	7.3	3.9
184	2	-53.4	-43.5	38.8	Supramarginal Gyrus: Posterior	6.83	3.65
182	2	-42	-65.6	41.7	Lateral Occipital Cortex: Superior	6.43	3.44
174	5	-7.4	-34.1	67.5	Postcentral Gyrus	6.18	3.3
47	2	54.2	-45.2	36.9	Supramarginal Gyrus: Posterior	6.15	3.29
64	1	56.5	-8.5	-14.3	Middle Temporal Gyrus: Posterior	6.1	3.26
14	2	40.7	14.5	48.2	Middle Frontal Gyrus	5.99	3.2
17	2	33.4	37.3	-16.4	Frontal Pole	5.96	3.19
183	1	-51.4	-56.3	20.5	Angular Gyrus	5.95	3.18
149	1	-39.3	17.2	46.7	Middle Frontal Gyrus	5.9	3.15
157	2	-46.2	7.9	28.6	Precentral Gyrus	5.87	3.14
185	1	-38	6.1	-37.9	Temporal Pole	5.84	3.12
43	8	31.6	-60.8	49.2	Lateral Occipital Cortex: Superior	5.82	3.11
200	8	-42.6	-52.1	-17.4	Temporal Occipital Fusiform Cortex	5.82	3.11
241	7	-40.3	-74.2	-29.1	Cerebellum	5.39	2.88
29	4	13.7	6.3	65.4	Superior Frontal Gyrus	5.37	2.87
234	4	-30.5	-23.9	-26.6	Parahippocampal Gyrus: Anterior	5.34	2.85
247	2	-10.3	-81.7	-32.3	Cerebellum	5.23	2.8
187	1	-49.5	11.1	-30.6	Temporal Pole	5.09	2.72
252	4	-46.4	-46.8	-42.9	Cerebellum	5	2.67

31	2	39.7	3.4	34	Precentral Gyrus	4.84	2.59
3	3	5.1	34.9	-17.4	Frontal Medial Cortex	4.74	2.53
104	4	23.5	-35.9	-43	Cerebellum	4.59	2.45
19	2	48.3	35.7	15.1	Frontal Pole	4.42	2.37
41	8	25.2	-52.4	68.1	Superior Parietal Lobule	4.35	2.33
209	8	-48.3	-67.4	1.1	Lateral Occipital Cortex: Inferior	4.31	2.3
5	3	8.2	45.9	-1.7	Paracingulate Gyrus	4.29	2.3
145	1	-10.1	55.7	30.2	Frontal Pole	4.17	2.23
48	2	47.8	-61.6	34.7	Lateral Occipital Cortex: Superior	4.14	2.22
175	8	-25.4	-54.7	64	Superior Parietal Lobe	4	2.14
61	5	59.2	-3.4	2.7	Planum Polare	3.96	2.12
220	4	-3.8	-5	32.6	Cingulate Gyrus: Anterior	3.84	2.05
140	1	-6	48.1	11.7	Paracingulate Gyrus	3.7	1.98
37	5	38.3	-12.5	-1.1	Insular Cortex	3.58	1.91
24	5	6	-22.3	65.6	Precentral Gyrus	3.5	1.87
147	2	-46.1	28.2	26.8	Middle Frontal Gyrus	3.46	1.85
197	5	-57	-14.5	-6.9	Superior Temporal Gyrus: Posterior	3.46	1.85
150	1	-5	17.7	46.1	Paracingulate Gyrus	3.37	1.8
121	4	12.7	12.9	11.5	Caudate	3.22	1.72
258	4	-12.5	11.6	8.7	Caudate	3.21	1.72
80	6	7.8	-88.6	11.9	Occipital Lobe	3.19	1.71
134	3	-5.4	29.1	-10.1	Subcallosal Cortex	3.15	1.69
177	8	-28.4	-62.3	40.4	Lateral Occipital Cortex: Superior	3.13	1.67
262	4	-9.6	-25.4	-1.4	Thalamus	3.11	1.66
1	2	13.9	56.8	-16.6	Frontal Pole	3.1	1.66

250	4	-22.7	-57.9	-48.8	Cerebellum	3.08	1.65
54	1	50	-33.8	-0.7	Middle Temporal Gyrus: Posterior	3.07	1.64
256	4	-24.3	-37.8	-44.3	Cerebellum	3.04	1.63
179	5	-35.7	-39.3	47.7	Superior Parietal Lobe	3.02	1.61
237	4	-8.7	-50.6	-39.6	Cerebellum	3.01	1.61

Supplementary Table 17. Sensitivity analysis varying prior distributions: Social Cognitive Measures predicting Communicative success.

**Model**	**BF_{half} (r = 0.18)**	**BF_{wide} (r = 0.5)**	**$BF_{extrawide}$ (r = 0.71)**
Confederate	6.67	6.67	6.67
IRI + Confederate	5.32	5.35	5.31
MRMS + Confederate	3.81	3.8	3.84
FaceTask + IRI + Confederate	2.14	2.14	2.14
FaceTask + Confederate	2.1	2.1	2.1
IRI + MRMS + Confederate	2.09	2.07	2.06
EA + IRI + Confederate	1.96	1.95	1.95
EA + Confederate	1.84	1.84	1.83
FaceTask + MRMS + Confederate	1.56	1.56	1.57
EA + MRMS + Confederate	1.44	1.44	1.44
FaceTask + IRI + MRMS + Confederate	0.99	0.99	0.99
FaceTask + EA + IRI + Confederate	0.94	0.95	0.94
EA + IRI + MRMS +	0.92	0.92	0.91

Confederate			
FaceTask + EA + Confederate	0.82	0.81	0.82
FaceTask + EA + MRMS + Confederate	0.7	0.69	0.7
FaceTask + EA + IRI + MRMS + Confederate	0.49	0.49	0.49

Supplementary Table 18. Sensitivity analysis varying prior distributions: Similarity Measures predicting Communicative Success.

Model	BF_{half} (r = 0.18)	BF_{wide} (r = 0.5)	$BF_{extrawide}$ (r = 0.71)
Neural Similarity + Covariates	6.17	6.15	6.15
Activity Similarity + Neural Similarity + Covariates	3.63	3.62	3.62
Perceived Similarity + Neural Similarity + Covariates	3.03	3.02	3.02
Personality Similarity + Neural Similarity + Covariates	2.71	2.70	2.71
Covariates	1.88	1.88	1.88
Activity Similarity + Perceived Similarity + Neural Similarity + Covariates	1.78	1.79	1.78
Activity Similarity + Personality Similarity +	1.73	1.73	1.73

Neural Similarity + Covariates			
Personality Similarity + Perceived Similarity + Neural Similarity + Covariates	1.44	1.44	1.44
Activity Similarity + Covariates	1.30	1.30	1.30
Activity Similarity + Personality Similarity + Perceived Similarity + Neural Similarity + Covariates	0.92	0.92	0.92
Personality Similarity + Covariates	0.89	0.89	0.89
Perceived Similarity + Covariates	0.85	0.85	0.85
Activity Similarity + Personality Similarity + Covariates	0.69	0.69	0.69
Activity Similarity + Perceived Similarity + Covariates	0.64	0.63	0.64
Personality Similarity + Perceived Similarity + Covariates	0.45	0.45	0.45
Activity Similarity + Personality Similarity + Perceived Similarity + Covariates	0.36	0.37	0.36

Supplementary Table 19. Sensitivity analysis varying prior distributions: Social Cognitive Measures predicting Perceived Interaction Quality.

Model	BF_{half} (r = 0.18)	BF_{wide} (r = 0.5)	$BF_{extrawide}$ (r = 0.71)
MRMS + Confederate	2.28	2.29	2.28
EA + MRMS + Confederate	0.77	0.77	0.77
IRI + MRMS + Confederate	0.76	0.75	0.75
FaceTask + MRMS + Confederate	0.74	0.74	0.74
IRI + Confederate	0.38	0.38	0.38
EA + IRI + MRMS + Confederate	0.32	0.32	0.32
FaceTask + EA + MRMS + Confederate	0.31	0.31	0.31
FaceTask + IRI + MRMS + Confederate	0.30	0.30	0.30
Confederate	0.20	0.20	0.20
EA + IRI + Confederate	0.17	0.17	0.17
FaceTask + EA + IRI + MRMS + Confederate	0.15	0.15	0.15
FaceTask + IRI + Confederate	0.14	0.14	0.14
FaceTask + EA + IRI + Confederate	0.08	0.08	0.08
FaceTask + Confederate	0.06	0.06	0.06
EA + Confederate	0.06	0.06	0.06
FaceTask + EA + Confederate	0.03	0.03	0.03

Supplementary Table 20. Sensitivity analysis varying prior distributions: Similarity Measures predicting Perceived Interaction Quality.

Model	BF_{half} (r = 0.18)	BF_{wide} (r = 0.5)	$BF_{extrawide}$ (r = 0.71)
Perceived Similarity + Covariates	259.12	259.03	260.46
Activity Similarity + Perceived Similarity + Covariates	92.61	92.36	92.42
Perceived Similarity + Neural Similarity + Covariates	89.83	90.00	89.76
Personality Similarity + Perceived Similarity + Covariates	81.51	81.92	81.31
Activity Similarity + Perceived Similarity + Neural Similarity + Covariates	35.87	35.75	35.85
Activity Similarity + Personality Similarity + Perceived Similarity + Covariates	32.78	32.95	32.77
Personality Similarity + Perceived Similarity + Neural Similarity + Covariates	31.50	31.65	31.62
Activity Similarity + Personality Similarity +	13.89	13.92	13.94

Perceived Similarity + Neural Similarity + Covariates			
Covariates	0.03	0.03	0.03
Activity Similarity + Covariates	0.02	0.02	0.02
Activity Similarity + Neural Similarity + Covariates	0.02	0.02	0.02
Neural Similarity + Covariates	0.02	0.02	0.02
Personality Similarity + Covariates	0.01	0.01	0.01
Activity Similarity + Personality Similarity + Covariates	0.01	0.01	0.01
Activity Similarity + Personality Similarity + Neural Similarity + Covariates	0.01	0.01	0.01
Personality Similarity + Neural Similarity + Covariates	0.01	0.01	0.01

Supplementary Table 21. Exploratory Interaction Models: Perceived Interaction Quality

Analysis comparing the interaction of each similarity measure (perceived similarity, activity similarity, personality similarity, neural similarity) and mind-reading motivation (MRMS) on perceived interaction quality.

Model	BF	BF_{model} / $BF_{covariates}$
MRMS + Perceived Similarity + Covariates	387.73	14866.08

Perceived Similarity + Covariates	259.66	9955.88
MRMS + Perceived Similarity + Activity Similarity + Covariates	211.52	8110.11
MRMS + Perceived Similarity + MRMS:Perceived Similarity + Covariates	152.50	5846.99
MRMS + Perceived Similarity + Neural Similarity + Covariates	148.79	5705.02
MRMS + Perceived Similarity + Personality Similarity + Covariates	135.10	5179.76
MRMS + Perceived Similarity + Activity Similarity + MRMS:Activity Similarity + Covariates	103.82	3980.68
MRMS + Perceived Similarity + Activity Similarity + Neural Similarity + Covariates	93.87	3599.03
Perceived Similarity + Activity Similarity + Covariates	92.43	3544.03
MRMS + Perceived Similarity + MRMS:Perceived Similarity + Activity Similarity + Covariates	91.91	3523.95
Perceived Similarity + Neural Similarity + Covariates	90.43	3467.28
Perceived Similarity + Personality Similarity + Covariates	81.63	3129.64
MRMS + Perceived Similarity + Activity Similarity + Personality Similarity + Covariates	79.06	3031.33
MRMS + Perceived Similarity + Neural Similarity + MRMS:Neural Similarity + Covariates	75.01	2876.12
MRMS + Perceived Similarity + MRMS:Perceived Similarity + Neural Similarity + Covariates	62.69	2403.46
MRMS + Perceived Similarity + Personality Similarity + MRMS:Personality Similarity + Covariates	60.40	2315.88
MRMS + Perceived Similarity + MRMS:Perceived Similarity + Personality Similarity + Covariates	59.91	2297.14
MRMS + Perceived Similarity + MRMS:Perceived Similarity + Activity Similarity + MRMS:Activity Similarity + Covariates	57.88	2219.31
MRMS + Perceived Similarity + Personality Similarity + Neural Similarity + Covariates	55.14	2114.02

MRMS + Perceived Similarity + Activity Similarity + MRMS:Activity Similarity + Neural Similarity + Covariates	48.44	1857.24
MRMS + Perceived Similarity + Activity Similarity + Neural Similarity + MRMS:Neural Similarity + Covariates	42.87	1643.58
MRMS + Perceived Similarity + MRMS:Perceived Similarity + Activity Similarity + Neural Similarity + Covariates	42.22	1618.66
MRMS + Perceived Similarity + Activity Similarity + MRMS:Activity Similarity + Personality Similarity + Covariates	40.57	1555.35
MRMS + Perceived Similarity + MRMS:Perceived Similarity + Activity Similarity + Personality Similarity + Covariates	37.85	1451.31
MRMS + Perceived Similarity + MRMS:Perceived Similarity + Neural Similarity + MRMS:Neural Similarity + Covariates	37.64	1443.21
MRMS + Perceived Similarity + Activity Similarity + Personality Similarity + Neural Similarity + Covariates	36.58	1402.49
Perceived Similarity + Activity Similarity + Neural Similarity + Covariates	35.89	1376.09
MRMS + Perceived Similarity + Activity Similarity + Personality Similarity + MRMS:Personality Similarity + Covariates	35.16	1348.25
Perceived Similarity + Activity Similarity + Personality Similarity + Covariates	32.77	1256.41
Perceived Similarity + Personality Similarity + Neural Similarity + Covariates	31.54	1209.30
MRMS + Perceived Similarity + Activity Similarity + MRMS:Activity Similarity + Neural Similarity + MRMS:Neural Similarity + Confederate + Number of Runs + FD Similarity	29.56	1133.24
MRMS + Perceived Similarity + Personality Similarity + Neural Similarity + MRMS:Neural Similarity + Covariates	29.46	1129.64

MRMS + Perceived Similarity + MRMS:Perceived Similarity + Activity Similarity + MRMS:Activity Similarity + Neural Similarity + Confederate + Number of Runs + FD Similarity	27.58	1057.31
MRMS + Perceived Similarity + MRMS:Perceived Similarity + Personality Similarity + MRMS:Personality Similarity + Covariates	26.31	1008.61
MRMS + Perceived Similarity + Personality Similarity + MRMS:Personality Similarity + Neural Similarity + Covariates	26.30	1008.51
MRMS + Perceived Similarity + MRMS:Perceived Similarity + Personality Similarity + Neural Similarity + Covariates	25.92	993.99
MRMS + Perceived Similarity + MRMS:Perceived Similarity + Activity Similarity + MRMS:Activity Similarity + Neural Similarity + MRMS:Neural Similarity + Covariates	24.89	954.47
MRMS + Perceived Similarity + MRMS:Perceived Similarity + Activity Similarity + MRMS:Activity Similarity + Personality Similarity + Confederate + Number of Runs + FD Similarity	24.78	950.18
MRMS + Perceived Similarity + Activity Similarity + MRMS:Activity Similarity + Personality Similarity + MRMS:Personality Similarity + Confederate + Number of Runs + FD Similarity	22.45	860.93
MRMS + Perceived Similarity + MRMS:Perceived Similarity + Activity Similarity + Neural Similarity + MRMS:Neural Similarity + Confederate + Number of Runs + FD Similarity	22.16	849.62
MRMS + Perceived Similarity + Activity Similarity + MRMS:Activity Similarity + Personality Similarity + Neural Similarity + Confederate + Number of Runs + FD Similarity	19.78	758.46
MRMS + Perceived Similarity + MRMS:Perceived Similarity + Activity Similarity + Personality Similarity + Neural Similarity + Confederate + Number of Runs + FD Similarity	17.97	689.01

MRMS + Perceived Similarity + Activity Similarity + Personality Similarity + Neural Similarity + MRMS:Neural Similarity + Confederate + Number of Runs + FD Similarity	17.67	677.55
MRMS + Perceived Similarity + Activity Similarity + Personality Similarity + MRMS:Personality Similarity + Neural Similarity + Confederate + Number of Runs + FD Similarity	16.94	649.57
MRMS + Perceived Similarity + MRMS:Perceived Similarity + Activity Similarity + Personality Similarity + MRMS:Personality Similarity + Confederate + Number of Runs + FD Similarity	16.67	639.26
MRMS + Perceived Similarity + MRMS:Perceived Similarity + Personality Similarity + Neural Similarity + MRMS:Neural Similarity + Confederate + Number of Runs + FD Similarity	16.33	626.04
Perceived Similarity + Activity Similarity + Personality Similarity + Neural Similarity + Covariates	13.87	531.68
MRMS + Perceived Similarity + Personality Similarity + MRMS:Personality Similarity + Neural Similarity + MRMS:Neural Similarity + Confederate + Number of Runs + FD Similarity	13.16	504.72
MRMS + Perceived Similarity + MRMS:Perceived Similarity + Activity Similarity + MRMS:Activity Similarity + Personality Similarity + MRMS:Personality Similarity + Covariates	12.72	487.79
MRMS + Perceived Similarity + Activity Similarity + MRMS:Activity Similarity + Personality Similarity + Neural Similarity + MRMS:Neural Similarity + Covariates	12.51	479.72
MRMS + Perceived Similarity + MRMS:Perceived Similarity + Personality Similarity + MRMS:Personality Similarity + Neural Similarity + Confederate + Number of Runs + FD Similarity	12.09	463.53

MRMS + Perceived Similarity + MRMS:Perceived Similarity + Activity Similarity + MRMS:Activity Similarity + Personality Similarity + Neural Similarity + Covariates	12.08	463.14
MRMS + Perceived Similarity + Activity Similarity + MRMS:Activity Similarity + Personality Similarity + MRMS:Personality Similarity + Neural Similarity + Covariates	11.21	429.63
MRMS + Perceived Similarity + MRMS:Perceived Similarity + Activity Similarity + MRMS:Activity Similarity + Personality Similarity + Neural Similarity + MRMS:Neural Similarity + Covariates	11.15	427.38
MRMS + Perceived Similarity + MRMS:Perceived Similarity + Activity Similarity + Personality Similarity + Neural Similarity + MRMS:Neural Similarity + Covariates	9.90	379.69
MRMS + Perceived Similarity + MRMS:Perceived Similarity + Activity Similarity + Personality Similarity + MRMS:Personality Similarity + Neural Similarity + Covariates	8.21	314.76
MRMS + Perceived Similarity + Activity Similarity + Personality Similarity + MRMS:Personality Similarity + Neural Similarity + MRMS:Neural Similarity + Covariates	8.12	311.41
MRMS + Perceived Similarity + MRMS:Perceived Similarity + Personality Similarity + MRMS:Personality Similarity + Neural Similarity + MRMS:Neural Similarity + Covariates	7.08	271.58
MRMS + Perceived Similarity + Activity Similarity + MRMS:Activity Similarity + Personality Similarity + MRMS:Personality Similarity + Neural Similarity + MRMS:Neural Similarity + Covariates	6.58	252.23
MRMS + Perceived Similarity + MRMS:Perceived Similarity + Activity Similarity + MRMS:Activity Similarity + Personality Similarity + MRMS:Personality Similarity + Neural Similarity + Covariates	6.43	246.71

MRMS + Perceived Similarity + MRMS:Perceived Similarity + Activity Similarity + MRMS:Activity Similarity + Personality Similarity + MRMS:Personality Similarity + Neural Similarity + MRMS:Neural Similarity + Confederate + Number of Runs + FD Similarity	5.24	200.88
MRMS + Perceived Similarity + MRMS:Perceived Similarity + Activity Similarity + Personality Similarity + MRMS:Personality Similarity + Neural Similarity + MRMS:Neural Similarity + Covariates	4.46	171.02
MRMS + Activity Similarity + Neural Similarity + Covariates	0.48	18.53
MRMS + Activity Similarity + Covariates	0.41	15.89
MRMS + Activity Similarity + MRMS:Activity Similarity + Neural Similarity + Covariates	0.38	14.73
MRMS + Activity Similarity + MRMS:Activity Similarity + Covariates	0.34	13.04
MRMS + Activity Similarity + MRMS:Activity Similarity + Personality Similarity + MRMS:Personality Similarity + Covariates	0.26	10.13
MRMS + Activity Similarity + MRMS:Activity Similarity + Personality Similarity + MRMS:Personality Similarity + Neural Similarity + Confederate + Number of Runs + FD Similarity	0.25	9.53
MRMS + Activity Similarity + Personality Similarity + Neural Similarity + Covariates	0.22	8.51
MRMS + Activity Similarity + Neural Similarity + MRMS:Neural Similarity + Covariates	0.22	8.41
MRMS + Covariates	0.21	7.98
MRMS + Activity Similarity + Personality Similarity + MRMS:Personality Similarity + Neural Similarity + Covariates	0.20	7.59
MRMS + Activity Similarity + MRMS:Activity Similarity + Neural Similarity + MRMS:Neural Similarity + Covariates	0.19	7.39
MRMS + Activity Similarity + Personality Similarity + MRMS:Personality Similarity + Covariates	0.19	7.37

MRMS + Activity Similarity + Personality Similarity + Covariates	0.19	7.19
MRMS + Activity Similarity + MRMS:Activity Similarity + Personality Similarity + Neural Similarity + Covariates	0.19	7.17
MRMS + Neural Similarity + Covariates	0.16	6.26
MRMS + Activity Similarity + MRMS:Activity Similarity + Personality Similarity + Covariates	0.16	6.17
MRMS + Personality Similarity + MRMS:Personality Similarity + Covariates	0.13	5.08
MRMS + Activity Similarity + MRMS:Activity Similarity + Personality Similarity + MRMS:Personality Similarity + Neural Similarity + MRMS:Neural Similarity + Covariates	0.13	4.80
MRMS + Activity Similarity + Personality Similarity + Neural Similarity + MRMS:Neural Similarity + Covariates	0.11	4.11
MRMS + Activity Similarity + Personality Similarity + MRMS:Personality Similarity + Neural Similarity + MRMS:Neural Similarity + Confederate + Number of Runs + FD Similarity	0.10	3.90
MRMS + Personality Similarity + MRMS:Personality Similarity + Neural Similarity + Covariates	0.10	3.83
MRMS + Activity Similarity + MRMS:Activity Similarity + Personality Similarity + Neural Similarity + MRMS:Neural Similarity + Confederate + Number of Runs + FD Similarity	0.10	3.80
MRMS + Personality Similarity + Covariates	0.09	3.51
MRMS + Neural Similarity + MRMS:Neural Similarity + Covariates	0.08	2.99
MRMS + Personality Similarity + Neural Similarity + Covariates	0.08	2.89
MRMS + Personality Similarity + MRMS:Personality Similarity + Neural Similarity + MRMS:Neural Similarity + Covariates	0.05	1.89
MRMS + Personality Similarity + Neural Similarity + MRMS:Neural Similarity + Covariates	0.04	1.48

Covariates	0.03	1.00
Activity Similarity + Covariates	0.02	0.85
Activity Similarity + Neural Similarity + Covariates	0.02	0.72
Neural Similarity + Covariates	0.02	0.69
Personality Similarity + Covariates	0.01	0.49
Activity Similarity + Personality Similarity + Covariates	0.01	0.45
Activity Similarity + Personality Similarity + Neural Similarity + Covariates	0.01	0.44

Supplementary References

Abraham, A., Pedregosa, F., Eickenberg, M., Gervais, P., Mueller, A., Kossaifi, J., Gramfort, A., Thirion, B., & Varoquaux, G. (2014). Machine learning for neuroimaging with Scikit-Learn. *Frontiers in Neuroinformatics, 8,* 14. https://doi.org/10.3389/fninf.2014.00014

Avants, B. B., Epstein, C. L., Grossman, M., & Gee. J. C. (2008). Symmetric diffeomorphic image registration with cross-correlation: Evaluating automated labeling of elderly and neurodegenerative brain. *Medical Image Analysis, 12*(1), 26–41. https://doi.org/10.1016/j.media.2007.06.004

Behzadi, Y., Restom, K., Liau, J., & Liu, T. T. (2007). A component based noise correction method (CompCor) for BOLD and perfusion based fMRI. *NeuroImage, 37*(1), 90–101. https://doi.org/10.1016/j.neuroimage.2007.04.042

Carpenter, J. M., Green, M. C., & Vacharkulksemsuk, T. (2016). Beyond perspective-taking: Mind-reading motivation. *Motivation and Emotion, 40*(3), 358–374. https://doi.org/10.1007/s11031-016-9544-z

Cox, R. W., & Hyde, J. S. (1997). Software tools for analysis and visualization of fMRI data. *NMR in Biomedicine, 10*(4-5), 171–78. https://doi.org/10.1002/(SICI)1099-1492(199706/08)10:4/5<171::AID-NBM453>3.0.CO;2-L

Dale, A. M., Fischl, B., & Sereno, M. I. (1999). Cortical surface-based analysis: I. Segmentation and surface reconstruction. *NeuroImage, 9*(2), 179–94. https://doi.org/10.1006/nimg.1998.0395

Davis, M. H. (1980). A multidimensional approach to individual differences in empathy. *JSAS Catalog of Selected Documents in Psychology, 10,* 85.

Davis, M. H. (1983). Measuring individual differences in empathy: Evidence for a multidimensional approach. *Journal of Personality and Social Psychology, 44*(1), 113–126. https://doi.org/10.1037/0022-3514.44.1.113

Esteban, O., Blair, R., Markiewicz, C. J., Berleant, S. L., Moodie, C., Ma, F., Isik, A. I., et al. (2018). FMRIPrep. Zenodo. https://doi.org/10.5281/zenodo.852659

Esteban, O., Markiewicz, C. J., Blair, R. W., Moodie, C. A., Isik, A. I., Erramuzpe, A., Kent, J., Goncalves, M., DuPre, E., Snyder, M., Oya, H., Ghosh, S. S., Wright, J., Durnez, J., Poldrack, R. A, & Gorgolewskki, K. J. (2018). "fMRIPrep: A robust preprocessing pipeline for functional MRI." *Nature Methods, 16,* 111–116. https://doi.org/10.1038/s41592-018-0235-4

Evans, A. C., Janke, A. L., Collins, D., L., & Baillet, S. (2012). Brain templates and atlases. *NeuroImage, 62*(2), 911–22. https://doi.org/10.1016/j.neuroimage.2012.01.024

Fonov, V.S., Evans, A.C., McKinstry, R.C., Almli, C.R., & Collins, D.L. (2009). Unbiased nonlinear average age-appropriate brain templates from birth to adulthood. *NeuroImage, 47,* S102. https://doi.org/10.1016/S1053-8119(09)70884-5

Glasser, M. F., Sotiropoulos, S. N., Wilson, J. A., Coalson, T. S., Fischl, B., Andersson, J. L., Xu, J., et al. (2013). The minimal preprocessing pipelines for the Human Connectome Project. *NeuroImage, 80,* 105–24. https://doi.org/10.1016/j.neuroimage.2013.04.127

Golan, O., Baron-Cohen, S., & Hill, J. (2006). The Cambridge Mindreading (CAM) face-voice battery: Testing complex emotion recognition in adults with and without Asperger Syndrome. *Journal of Autism and Developmental Disorders, 36*(2), 169–183. https://doi.org/10.1007/s10803-005-0057-y

Goldberg, L. R. (1992). The development of markers for the Big-Five factor structure. *Psychological Assessment, 4,* 26-42.

Gorgolewski, K., Burns, C. D., Madison, C., Clark, D., Halchenko, Y. O., Waskom, M. L., & Ghosh. S. (2011). Nipype: A flexible, lightweight and extensible neuroimaging data processing framework in Python. *Frontiers in Neuroinformatics, 5,* 13. https://doi.org/10.3389/fninf.2011.00013

Gorgolewski, K. J., Esteban, O., Markiewicz, C. J., Ziegler, E., Ellis, D. G., Notter, M. P., Jarecka, D., et al. (2018). Nipype. Zenodo. https://doi.org/10.5281/zenodo.596855

Greve, D. N., & Fischl, B. (2009). Accurate and robust brain image alignment using boundary-based registration. *NeuroImage 48*(1), 63–72. https://doi.org/10.1016/j.neuroimage.2009.06.060

Jenkinson, M., Bannister, P., Brady, M., & Smith, S. (2002). Improved optimization for the robust and accurate linear registration and motion correction of brain images. *NeuroImage, 17*(2), 825–41. https://doi.org/10.1006/nimg.2002.1132

Klein, A., Ghosh, S. S., Bao, F. S., Giard, J., Häme, Y., Stavsky, E., Lee, N., et al. (2017). Mindboggling morphometry of human brains. *PLOS Computational Biology, 13*(2), e1005350. https://doi.org/10.1371/journal.pcbi.1005350

Lanczos, C. (1964). Evaluation of noisy data. *Journal of the Society for Industrial and Applied Mathematics Series B Numerical Analysis, 1*(1), 76–85. https://doi.org/10.1137/0701007

McManus, I. C., Jonvik, H., Richards, P., & Paice, E. (2011). Vocation and avocation: Leisure activities correlate with professional engagement, but not burnout, in a cross-sectional survey of UK doctors. *BMC Medicine, 9*(1), 100. https://doi.org/10.1186/1741-7015-9-100

Ong, D. C., Wu, Z., Zhi-Xuan, T., Reddan, M., Kahhale, I., Mattek, A., & Zaki, J. (2021). Modeling emotion in complex stories: The Stanford Emotional Narratives Dataset. *IEEE Transactions on Affective Computing, 12*(3), 579–594. https://doi.org/10.1109/TAFFC.2019.2955949

Peirce, J. W., Gray, J. R., Simpson, S., MacAskill, M. R., Höchenberger, R., Sogo, H., Kastman, E., Lindeløv, J. (2019). PsychoPy2: Experiments in behavior made easy. *Behavior Research Methods, 51*, 195-203. https://doi.org/10.3758/s13428-018-01193-y

Power, J. D., Mitra, A., Laumann, T. O., Snyder, A. Z., Schlaggar, B. L., & Petersen, S. E. (2014). Methods to detect, characterize, and remove motion artifact in resting state fMRI. *NeuroImage, 84,* 320–41. https://doi.org/10.1016/j.neuroimage.2013.08.048

Pruim, R. H. R., Mennes, M., van Rooij, D., Llera, A., Buitelaar, J. K., & Beckmann, C. F. (2015). ICA-AROMA: A robust ICA-based strategy for removing motion artifacts from fMRI data. *NeuroImage, 112,* 267–77. https://doi.org/10.1016/j.neuroimage.2015.02.064

Satterthwaite, T. D., Elliott, M. A., Gerraty, R. T., Ruparel, K., Loughead, J., Calkins, M. E., Eickhoff, S. B., et al. (2013). An improved framework for confound regression and filtering for control of motion artifact in the preprocessing of resting-state functional connectivity data. *NeuroImage, 64*(1), 240–56. https://doi.org/10.1016/j.neuroimage.2012.08.052

Saucier, G. (1994). Mini-Markers: A brief version of Goldberg's unipolar Big-Five markers. Journal of Personality Assessment, 63(3), 506–516. https://doi.org/10.1207/s15327752jpa6303_8

Tustison, N. J., Avants, B. B., Cook, P. A., Zheng, Y., Egan, A., Yushkevich, P. A., & Gee, J. C. (2010). N4ITK: Improved N3 bias correction. *IEEE Transactions on Medical Imaging, 29*(6), 1310–20. https://doi.org/10.1109/TMI.2010.2046908

Zhang, Y., Brady, M., & Smith, S. (2001). Segmentation of brain MR images through a hidden Markov random field model and the expectation-maximization algorithm. *IEEE Transactions on Medical Imaging, 20*(1), 45–57. https://doi.org/10.1109/42.906424
